# Mesenchymal stem cell-derived conditioned medium demonstrates novel antibacterial effects in ocular bacterial infections

**DOI:** 10.1128/iai.00697-25

**Published:** 2026-04-30

**Authors:** Sairam Abbireddy, Joveeta Joseph, Bhupesh Bagga, Sachin Shukla

**Affiliations:** 1 Prof. Brien Holden Eye Research Centre, Hyderabad Eye Research Foundation, L V Prasad Eye Institute 28592 , Hyderabad, India; 2 Sudhakar and Sreekanth Ravi Stem Cell Biology Laboratory, Centre for Ocular Regeneration, L V Prasad Eye Institute, Hyderabad, India; 3 Jhaveri Microbiology Centre, L V Prasad Eye Institute, Hyderabad, India; 4 The Ramoji Foundation Centre for Ocular Infections, L V Prasad Eye Institute, Hyderabad, India; 5 Shantilal Shanghvi Cornea Institute, L V Prasad Eye Institute, Hyderabad, India; University of Illinois Chicago, Chicago, Illinois, USA

**Keywords:** mesenchymal stem cells, bacterial keratitis, antibacterial, infection, immunomodulation, cornea, antimicrobial peptide

## Abstract

Bacterial keratitis is a potentially blinding corneal infection, with *Staphylococcus aureus* and *Pseudomonas aeruginosa* being the leading gram-positive and gram-negative pathogens, respectively. The increasing antimicrobial resistance among these pathogens necessitates the development of newer therapeutic approaches. This study aims to assess the therapeutic efficacy of mesenchymal stem cell-derived conditioned medium (MSC-CM) for ocular isolates of *S. aureus* and *P. aeruginosa*. The MSCs were derived from human adipose tissue, bone marrow, dental pulp, and umbilical cord. The antibacterial effects of MSC-CM were studied through zone of inhibition and colony-forming unit assays, whereas morphological changes were studied through scanning electron microscopy. Furthermore, to mimic bacterial infections, human corneal epithelial cells were stimulated with bacterial endotoxins, and changes in the expression of antimicrobial peptides (LL-37, Human Beta Defensin-3 [HBD-3], Dermcidin, Hepcidin-25, and Lipocalin-2) and inflammatory mediators (IL-6 and TNF-α) were evaluated through enzyme-linked immunosorbent assay. While HBD-3 was present in the highest concentration across all sources of MSCs, treatment with MSC-CM further upregulated the expression of antimicrobial peptides while downregulating the expression of pro-inflammatory mediators, suggesting its immunomodulatory effects. The results suggest that MSC-CM exhibits antibacterial effects against the tested ATCC strains and clinical isolates of *S. aureus* and *P. aeruginosa* causing ocular infections, with the highest efficacy shown by the adipose tissue-derived MSC-CM. Furthermore, in an *ex vivo* human corneal infection model, adipose-derived MSC-CM decreased the bacterial burden while preserving corneal transparency compared to untreated controls. These findings indicate the promising therapeutic potential of MSC-CM for ocular bacterial infections.

## INTRODUCTION

Bacterial keratitis is the most prevalent and serious form of ocular infection (1). If left untreated, it leads to vision impairment (2). The etiology and pathogenesis of bacterial keratitis are influenced by several factors, including contact lens use, corneal trauma, immunosuppressive therapy, and a history of ocular surgery, particularly corneal grafting (3–5). *Staphylococcus aureus* and *Pseudomonas aeruginosa* are the leading gram-positive and gram-negative bacterial pathogens in corneal infections, respectively, which are increasingly developing resistance to a wide range of antibiotics, including quinolones and aminoglycosides (6–11). With antibiotic resistance on the rise, there is an urgent need for newer therapeutic approaches in the management of keratitis to preserve vision and improve patient outcomes.

Mesenchymal stem cells (MSCs) derived from different tissue sources, including adipose tissue (AD) (12–14), bone marrow (BM) (15–19), dental pulp (DP) (20), and umbilical cord (UC) (16), have been reported to produce antimicrobial peptides (AMPs), including LL-37, Hepcidin, Dermcidin, and Lipocalin-2 (LCN-2), and exhibit antibacterial activity against pathogens such as *S. aureus, P. aeruginosa,* and *Escherichia coli* by disrupting bacterial cell membranes and inhibiting biofilm formation (21, 22). These therapeutic effects of MSCs have been demonstrated across various preclinical models, including sepsis, acute lung injury, cystic fibrosis, and chronic wound infections (14, 15, 23). Additionally, MSCs have also been investigated in diseases like rheumatoid arthritis, Alzheimer’s disease, and myocardial infarction for their tissue repair and immunomodulatory properties (24–26).

MSCs possess strong immunomodulatory properties, which are primarily mediated through the release of bioactive factors collectively known as the MSC secretome. These secreted molecules respond to inflammatory signals in the surrounding environment and help regulate immune responses. In particular, MSCs release mediators such as prostaglandin E2 and indoleamine 2,3-dioxygenase, which suppress pro-inflammatory cytokines, including IL-6 and TNF-α (27). These factors also promote the shift of macrophages from a pro-inflammatory (M1) phenotype to an anti-inflammatory (M2) phenotype and reduce the activation of effector T cells (28). Additionally, MSC-derived extracellular vesicles contain regulatory microRNAs that inhibit inflammatory pathways such as NF-κB signaling, thereby reducing inflammation and supporting tissue repair and immune balance (29). A recent study reported the antibacterial properties of AD-, BM-, and UC-MSC secretome for the *E. coli* strains isolated from urinary tract infections (30). However, the therapeutic application of MSCs in ocular bacterial infections, including bacterial keratitis, remains largely unexplored.

To the best of our knowledge, this study is the first to collectively investigate the antibacterial properties of AD-, BM-, DP-, and UC-MSC-conditioned medium (CM) against both the ATCC reference strains and clinical isolates of *S. aureus* and *P. aeruginosa* from patients with ocular infections and further analyze their AMPs and immunomodulatory properties against bacterial endotoxins.

## MATERIALS AND METHODS

### Cell culture and collection of conditioned medium

Human MSCs from adipose tissue (#R7788110, ThermoFisher Scientific, USA), bone marrow (#PT-2501, Lonza Bioscience, USA), dental pulp (Stemade Biotech Pvt. Ltd., India), and umbilical cord (a generous gift from Dr. Subha N. Rath, Indian Institute of Technology-Hyderabad) were procured and cultured as per the supplier’s instructions. Human corneal epithelial cells (HCECs) (#HCE-2 [50.B1] CRL-11135, isolated from the cornea of a male donor, deposited by CR Kahn, Gillette Medical Evaluation Laboratories, USA, cited in US Pat. No. 5672498) were procured and maintained without antibiotics as per the recommended protocol and our previous reports (19, 31). The MSCs were cultured in T-75 flasks (3 × 10^5^ cells/mL) using Dulbecco’s Modified Eagle Medium (DMEM; High Glucose, Cat. No. 11965-092, Gibco, USA) supplemented with 10% fetal bovine serum (FBS; Cat. No. A5256701, Gibco, USA). The MSC-CM was collected as per our previous publication (19). Briefly, when MSC cultures reached 75% confluency, the medium was gently replaced with serum-free DMEM to allow for the collection of conditioned medium. After 24 hours, the MSC-CM was collected, centrifuged at 2,000 rpm for 10 minutes to eliminate any remaining cells and debris, and filtered through a sterile 0.2 μm syringe filter (Millipore, USA). The MSC-CM, thus obtained, was then aliquoted and stored at −80°C for further experimental use. All MSCs and HCECs used for the experiments were utilized between passages 3 and 5.

### Bacterial culture

The ATCC strains and clinical isolates (isolated from the patients with ocular bacterial infections) of *S. aureus* (ATCC #29213, Clinical #L-1791/20) and *P. aeruginosa* (ATCC #27853, Clinical #L-2050/18) were characterized and identified using the VITEK-2 COMPACT system (bioMerieux, France) at the Jhaveri Microbiology Centre, L V Prasad Eye Institute, Hyderabad, India. The clinical isolate of *S. aureus* (#L-1791/20) displayed a methicillin-susceptible *S. aureus* phenotype, susceptible to oxacillin, vancomycin, linezolid, gentamicin, and tetracyclines, while resistant to azithromycin, penicillin, ciprofloxacin, levofloxacin, and clindamycin. In contrast, the clinical isolate of *P. aeruginosa* (#L-2050/18) was susceptible to β-lactams, carbapenems, aminoglycosides, and fluoroquinolones, with resistance limited to tigecycline. The ATCC strains of *S. aureus* (ATCC #29213) and *P. aeruginosa* (ATCC #27853) served as Clinical and Laboratory Standards Institute-recommended reference strains for antimicrobial susceptibility testing, with no strain-specific resistance profiles as per ATCC documentation.

The overnight cultures of the bacterial strains routinely grown in Mueller-Hinton Agar (MHA, #38210000, HiMedia, India) plates for 18 hours at 37°C were used for the experiments. The number of bacteria was calculated (DensiCHEK plus, BioMerieux, Germany) according to the following equation: McFarland unit = 1.0 corresponds to 3 × 10^8^ bacteria/mL, as described in reference 19.

### Zone of inhibition assay

The antibacterial activity of MSC-CM was evaluated using the Kirby-Bauer disk diffusion method (32). The above-mentioned ATCC and clinical isolates of *S. aureus* and *P. aeruginosa* were cultured overnight and adjusted to the 0.5 McFarland standard. The standardized inoculum was uniformly spread on Mueller-Hinton agar plates using sterile swabs. Sterile 6 mm (Cat# SD067, HiMedia, India) filter paper discs were aseptically placed on the MHA (#38210000, HiMedia, India) agar surface and loaded with 20 μL of each MSC-CM derived from AD-, BM-, DP-, and UC-MSCs, respectively. The DMEM and ciprofloxacin (0.3%)-loaded discs were used as negative and positive controls, respectively. Plates were incubated at 37°C for 24 hours, and zones of inhibition (ZOI) were quantified (in mm) using ImageJ software by measuring the diameter of the clear zone from the captured plate images.

### Scanning electron microscopy

Overnight-grown cultures of *S. aureus* and *P. aeruginosa* (ATCC and clinical isolates) were adjusted to a 2 McFarland standard and treated with 500 µL of MSC-CM for 24 hours. At the same time, DMEM-treated cultures served as the untreated control. After incubation, 100 μL of each sample was placed onto sterile glass coverslips and allowed to settle for 30 minutes at room temperature. Samples were fixed with 4% paraformaldehyde overnight at 4°C. After fixation, samples were dehydrated sequentially through a graded ethanol (10%, 30%, 50%, 70%, 90%, and 100%) and air-dried overnight. Thereafter, the coverslips were mounted on aluminum stubs using double-sided carbon tape and sputter-coated with a gold palladium layer to ensure surface conductivity, and imaging was performed using a scanning electron microscope (Carl Zeiss Model EVO 18; Carl Zeiss, Germany). Representative micrographs were captured at a magnification of 15,000× to observe surface morphological alterations.

### Colony-forming unit assay

The colony-forming unit (CFU) assay was performed, as previously described, to investigate the role of MSC-CM on the growth of *S. aureus* and *P. aeruginosa* (19). Bacterial suspensions of *S. aureus* or *P. aeruginosa*, each with 6 × 10⁸ CFU/mL, were prepared. In 12-well plates, 500 µL of MSC-CM from AD-, BM-, DP-, or UC-MSCs was introduced to the bacteria and co-incubated for 18 hours at 37°C. Thereafter, serial dilutions (log 10) were performed and plated onto Mueller-Hinton Agar (#38210000, HiMedia, India), followed by incubation for 24 hours at 37°C. Colonies were counted manually, and CFU/mL was determined. Control wells consisted of bacteria incubated in DMEM (500 µL) under the same conditions.

### Time-kill assay

A time-course CFU assay was performed to evaluate the kinetics of antibacterial activity of MSC-CM. Bacterial suspensions were incubated with AD-, BM-, DP-, or UC-MSC-CM or DMEM (control) at 37°C. At 2, 4, 6, 12, and 24 hours, aliquots were collected from independent wells, serially diluted (up to 10^−6^), and plated on MHA for CFU enumeration as described above. Undiluted samples were additionally plated at 2 and 24 hours for qualitative confirmation. Bacterial counts were expressed as log_10_ CFU/mL.

### Induction with bacterial endotoxins

HCECs were cultured in 12-well tissue culture plates at a 1 × 10^5^ cells/well concentration in antibiotic-free medium. At 75% confluency, 10 µg/mL of bacterial endotoxins: lipoteichoic acid (LTA) (Cat# L2515-5MG, Sigma Aldrich, USA), derived from *S. aureus,* or lipopolysaccharide (LPS) (Cat# L9143, Sigma-Aldrich, USA), derived from *P. aeruginosa,* was added to the HCECs and incubated at 37°C in a 5% CO_2_ incubator for 24 hours. After 24 hours, LTA/LPS was removed, and HCECs were further treated with respective MSC-CM for 24 hours. Supernatants (1 mL) were collected and stored at −80°C for enzyme-linked immunosorbent assay (ELISA).

### Enzyme-linked immunosorbent assay

First, the total protein concentration of the culture supernatants was quantified utilizing a NanoDrop2000c Spectrophotometer (Thermo Fisher Scientific, USA). Subsequently, the concentrations of AMPs: LL-37 (Cathelicidin), Dermcidin, Human Beta Defensin-3 (HBD-3), Hepcidin-25, and Lipocalin-2 in MSC-CM were determined using ELISA following the manufacturer’s protocol (Human Antibacterial Protein LL-37 ELISA, REF: KBH2197; Human Dermcidin ELISA, REF: KBH9909; Human beta Defensin 3 ELISA, REF: KBH3240; Human High Sensitive Hepcidin25 ELISA, REF: KBH3763; and Human Lipocalin 2 ELISA, REF: KBH1429, Krishgen Biosystems, Germany). In addition, the expression of pro-inflammatory cytokines IL-6 and TNF-α in LTA-/LPS-stimulated HCECs was quantified using specific ELISA kits (Human IL-6 ELISA, REF: KB1068; Human TNF-α ELISA, REF: KB1145, Krishgen Biosystems, Germany). For cytokine assays, all incubation steps were carried out at room temperature unless otherwise stated. In summary, 100 μL of standards or samples was dispensed into the individual wells of the ELISA plate. The plates were sealed and incubated for 80 minutes at 37°C. Following this, 100 μL of diluted detection antibody was added to each well, and plates were sealed and incubated for 1 hour at 37°C. Thereafter, 100 μL of streptavidin: HRP solution was added to each well, sealed, and incubated for 1 hour at 37°C. Subsequently, 100 μL of TMB substrate was added to each well and incubated for 15–20 minutes at 37°C. Upon completion of the incubation, 100 μL of stop solution was added to each well, and absorbance was recorded at 450 nm using a spectrophotometer (SpectraMax M3, Molecular Devices, Radnor, PA, USA).

### *Ex vivo* human cornea infection model

Human donor corneas derived from cadavers with no reported history of any infectious disease at the time of death, unsuitable for transplantation, were obtained from the in-house Ramayamma International Eye Bank, L V Prasad Eye Institute, Hyderabad, India. All procedures were performed under aseptic conditions in a biosafety laminar flow hood. Corneas were rinsed thoroughly with sterile phosphate-buffered saline (PBS) to remove residual storage medium. For surface sterilization, corneas were immersed in 10% povidone-iodine (PI) solution for 5 minutes at room temperature, followed by nine successive washes with sterile PBS to ensure complete removal of residual PI solution. Sterilized corneas were placed with the epithelial side down in a sterile mold and filled with sterile molten MHA to provide structural support (33). After solidification of agar (~10 minutes), the agar-supported corneas were transferred epithelial side up onto sterile MHA plates under non-submerged conditions.

A localized injury was created at the central corneal surface using a sterile 26-gauge needle to facilitate bacterial adherence. A bacterial suspension (*S. aureus*/*P. aeruginosa*) was prepared from overnight cultures and adjusted to the 2 McFarland standard. A 10 µL volume of the bacterial suspension containing either *S. aureus* or *P. aeruginosa* was inoculated onto the injured central corneal surface under aseptic conditions. Corneas were incubated at 37°C in a humidified incubator for 24 hours to allow establishment of infection. Thereafter, the corneas were assigned to experimental groups and treated topically with 20 µL of AD-MSC-CM or ciprofloxacin, or left untreated as infected controls. AD-MSC-CM was prioritized for the *ex vivo* study based on its consistently superior antibacterial and antibiofilm activity observed *in vitro*, including exhibition of higher basal and stimulated expression of AMPs, while effectively downregulating pro-inflammatory cytokines in LTA- and LPS-induced models, suggesting the greatest potential for managing established tissue infections. Treatments (AD-MSC-CM or ciprofloxacin) were applied at 2-hour intervals over a total duration of 24 hours. Corneas were maintained at 37°C in an incubator throughout the experiment. Images of the cornea were captured at 2-hour intervals to monitor the progression/resolution of the infection in response to the treatment.

### Analysis of corneal opacity

Corneal opacity was assessed using standardized digital imaging. Images were analyzed using ImageJ software. Briefly, images were converted to an 8-bit grayscale format, and a fixed circular region of interest was applied to the central cornea. Mean gray intensity values were measured to quantify corneal opacity.

### Quantification of bacterial burden

At the experimental endpoint (24 hours after treatment initiation), the supporting MHA was carefully removed from the corneas under aseptic conditions. The corneal buttons were then excised using sterile scissors and transferred into sterile tubes containing PBS with 0.001% Tween-20. The corneal tissues were homogenized by vigorous vortexing to ensure efficient recovery of adherent bacteria. Serial 10-fold dilutions were prepared in sterile PBS, and aliquots of 100 µL were plated onto MHA plates. Plates were incubated at 37°C for 24 hours, and bacterial colonies were enumerated. Bacterial burden was calculated and expressed as log_10_ CFU/mL. The limit of detection was defined as 7 log_10_ CFU/mL.

### Biofilm eradication assay

The ability of MSC-CM to disrupt pre-established biofilms was assessed using a crystal violet microtiter plate assay. Briefly, S. *aureus* ATCC (#29213) and a clinical isolate (#L-1791/20), as well as *P. aeruginosa* ATCC (#27853) and a clinical isolate (#L-2050/18), were grown overnight in Brain Heart Infusion (BHI) broth at 37°C. The bacterial suspension was adjusted to approximately 1 × 10⁶ CFU/mL, and 200 µL was added to each well of a sterile 96-well plate. Plates were incubated at 37°C for 24 hours under static conditions to allow biofilm formation. Following the incubation for 24 hours, wells were gently washed three times with sterile PBS to remove non-adherent cells. Subsequently, 200 µL of undiluted MSC-CM derived from AD-, BM-, DP-, or UC-MSCs, or ciprofloxacin (0.3%) as a positive control, was added to the respective wells. Untreated control wells received BHI medium only, while blank controls consisting of BHI or DMEM without bacterial inoculation were included to account for any background interference. Plates were incubated for an additional 24 hours at 37°C. Following treatment, wells were washed with PBS, fixed with 100% methanol, and stained with crystal violet for 15 minutes at room temperature, and excess stain was removed by washing. The bound crystal violet was solubilized using 95% ethanol, and absorbance was measured at 570 nm using a microplate reader. Data were expressed as the percentage of biofilm growth relative to untreated biofilm controls.

### Statistical analysis

Data are presented as mean ± standard deviation (SD). Statistical analyses were performed using GraphPad Prism. For comparisons involving multiple treatment groups against a single control, one-way ANOVA followed by Dunnett’s multiple treatment comparisons test was used. For comparisons among all treatment groups, one-way ANOVA followed by Tukey’s *post hoc* test was applied. For experiments involving two independent variables, such as treatment condition and bacterial strain (ATCC vs clinical isolate), two-way ANOVA followed by Tukey’s multiple comparisons test was performed. A *P*-value < 0.05 was considered statistically significant.

## RESULTS

### MSC-CM exhibits potent antibacterial activity against *S. aureus* and *P. aeruginosa*

**Fig 1:**
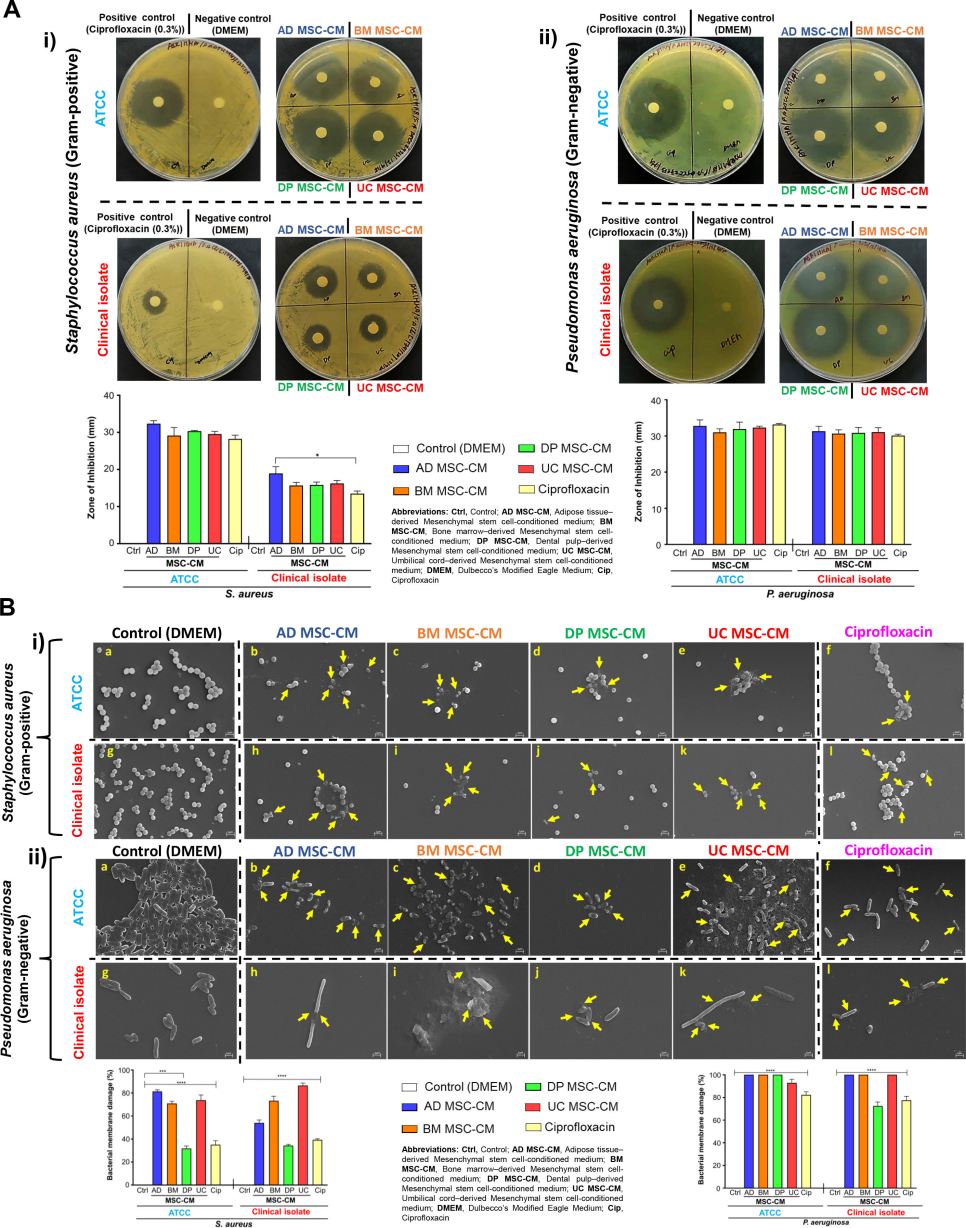
Antibacterial effects of MSC-CM on *S. aureus* and *P. aeruginosa*. (**A**) Zone of inhibition assay showing antibacterial activity of MSC-conditioned medium against (i) *S. aureus* ATCC (#29213) and clinical isolate (#L-1791/20), and (ii) *P. aeruginosa* ATCC (#27853) and clinical isolate (#L-2050/18). Sterile discs were loaded with 20 μL of MSC-CM, DMEM (negative control), or ciprofloxacin (0.3%, positive control) and placed on Mueller-Hinton agar plates inoculated with respective bacterial strains. Plates were incubated at 37°C for 24 hours, and inhibition zones were measured in millimeters using ImageJ. Data are presented as mean ± SD. Statistical analysis was performed using one-way ANOVA followed by Dunnett’s multiple comparisons test vs ciprofloxacin or control as indicated (*P < 0.05; **P < 0.01; ***P < 0.001; and ****P < 0.0001). (**B**) Scanning electron microscopy (SEM) analysis and quantification of bacterial membrane damage following 24-hour treatment with MSC-CM or ciprofloxacin in (i) *S. aureus* ATCC (#29213) and clinical isolate (#L-1791/20) and (ii) *P. aeruginosa* ATCC (#27853) and clinical isolate (#L-2050/18). Untreated controls (**a and g**) display intact bacterial morphology with smooth, preserved cell surfaces. In contrast, MSC-CM-treated bacteria (**b–e and h–k**) and ciprofloxacin-treated samples (positive control) (**f and l**) exhibit membrane disruption, cell wall damage, and surface irregularities (as indicated by yellow arrows). The percentage of membrane damage was quantified based on SEM morphological assessment of structurally compromised bacterial cells. Data are presented as mean ± SD. Statistical analysis was performed using one-way ANOVA followed by Dunnett’s multiple comparisons test vs ciprofloxacin or control as indicated (*P < 0.05; **P < 0.01; ***P < 0.001; and ****P < 0.0001). Samples were fixed in 4% paraformaldehyde, dehydrated through graded ethanol, air dried, sputter-coated with gold-palladium, and imaged using a Carl Zeiss EVO 18 SEM at 15,000× magnification. Scale bar = 1 µm.

The antibacterial potential of MSC-CM was evaluated against the ATCC and clinical isolates of *S. aureus* (Fig. 1A-i) and *P. aeruginosa* (Fig. 1A-ii) using the Kirby-Bauer disk diffusion method. Discs treated with MSC-CM exhibited clear ZOI, whereas DMEM (negative control) showed no inhibitory effect. Ciprofloxacin (0.3%) served as a positive control and produced distinct inhibition zones, validating the assay. For *S. aureus* ATCC (#29213), MSC-CM produced the following ZOI: AD-MSC-CM, 32.30 mm; BM-MSC-CM, 29.12 mm; DP-MSC-CM, 30.34 mm; and UC-MSC-CM, 29.50 mm, which were comparable to ciprofloxacin (28.22 mm), with no statistically significant differences observed (*P* > 0.05). For the clinical isolate (#L-1791/20), the ZOI were as follows: AD-MSC-CM, 18.92 mm; BM-MSC-CM, 15.72 mm; DP-MSC-CM, 15.82 mm; and UC-MSC-CM, 16.24 mm, while ciprofloxacin produced a smaller inhibition zone (13.48 mm). AD-MSC-CM showed significantly greater inhibition compared with ciprofloxacin (*P = 0.0116), whereas BM-, DP-, and UC-MSC-CM exhibited comparable antibacterial activity (*P* > 0.05). Importantly, Tukey’s multiple comparisons test revealed that both MSC-CM and ciprofloxacin produced significantly larger inhibition zones in the ATCC strain compared with the clinical isolate (*P* < 0.0001), indicating relatively lower susceptibility of the clinical strain (Fig. 1A-i).

Similarly, for *P. aeruginosa* ATCC strain (#27853), MSC-CM produced the following ZOI: AD-MSC-CM, 32.76 mm; BM-MSC-CM, 31.02 mm; DP-MSC-CM, 31.94 mm; and UC-MSC-CM: 32.33 mm, which were comparable to ciprofloxacin (33.14 mm), with no statistically significant differences between all four MSC-CM sources and ciprofloxacin (*P* > 0.05). The clinical isolate (#L-2050/18) also showed comparable inhibition zones (AD-MSC-CM, 31.33 mm; BM-MSC-CM, 30.67 mm; DP-MSC-CM, 30.85 mm; and UC-MSC-CM, 31.07 mm), including with ciprofloxacin treatment (30.09 mm), with no statistically significant differences observed (*P* > 0.05). Furthermore, no significant differences were detected between ATCC and clinical isolates treated with MSC-CM or ciprofloxacin treatments (*P* > 0.05), indicating similar susceptibility profiles (Fig. 1A-ii).

Collectively, these findings demonstrate that MSC-CM exhibits robust antibacterial activity against both the tested gram-positive (*S. aureus*) and gram-negative (*P. aeruginosa*) pathogens.

MSC-CM showed antibacterial efficacy comparable to ciprofloxacin in most conditions and demonstrated significantly greater activity than ciprofloxacin against the clinical isolate of *S. aureus* in the AD-MSC-CM-treated group (Fig. 1A-i).

### MSC-CM treatment induces morphological changes in *S. aureus* and *P. aeruginosa*

Scanning electron microscopy (SEM) revealed distinct morphological alterations in both *S. aureus* and *P. aeruginosa* (ATCC and clinical isolates) following treatment with MSC-CM or ciprofloxacin (Fig. 1B). Untreated control (DMEM-treated) cells of ATCC (Fig. 1B-a) and clinical isolate (Fig. 1B-g) of *S. aureus* (Fig. 1B-i) and *P. aeruginosa* (Fig. 1B-ii: a, g) showed smooth, intact, and well-preserved bacterial surfaces, characteristic of normal cellular morphology. In contrast, MSC-CM-treated bacterial cells of both *S. aureus* (Fig. 1B-i: b–e and h–k) and *P. aeruginosa* (Fig. 1B-ii: b–e and h–k) displayed clear structural abnormalities, including membrane collapse, surface irregularities, and cell wall disruption, as indicated by yellow arrows. Similar morphological alterations were observed in ciprofloxacin-treated bacteria (Fig. 1B-i: f, l and ii: f, l). Notably, cells of *P. aeruginosa* exhibited marked elongation and distortion (Fig. 1B-ii: h, k). To further substantiate these observations, quantitative analysis demonstrated a significant increase in the percentage of membrane-damaged cells following treatment with MSC-CM from various sources compared to untreated controls. In *S. aureus,* MSC-CM treatments against the ATCC (#29213) (AD, 81.32%; BM, 70.89%; DP, 31.83%; and UC, 73.73%) and clinical isolate (#1791/20) (AD, 54.07%; BM, 73.27%; DP, 34.27%; and UC, 86.50%) showed higher disruption than ciprofloxacin (ATCC, 35.02%; clinical isolate, 39.27%). Notably, in *P. aeruginosa,* MSC-CM against the ATCC (#27853) (AD, 100%; BM, 100%; DP, 100%; and UC, 92.83%) and clinical isolate (#L-2050/18) (AD, 100%; BM, 100%; DP, 72.5%; and UC, 100%) also outperformed ciprofloxacin (ATCC, 82.25%; clinical isolate, 77.5%). All treated groups demonstrated a marked increase in membrane compromise compared to the untreated controls. This supports the ability of MSC-CM to compromise bacterial cell envelope integrity. These findings correlate with the antibacterial effects observed in ZOI and CFU assays.

### MSC-CM inhibits the growth of the ATCC strain and the clinical isolate of *S. aureus*

**Fig 2:**
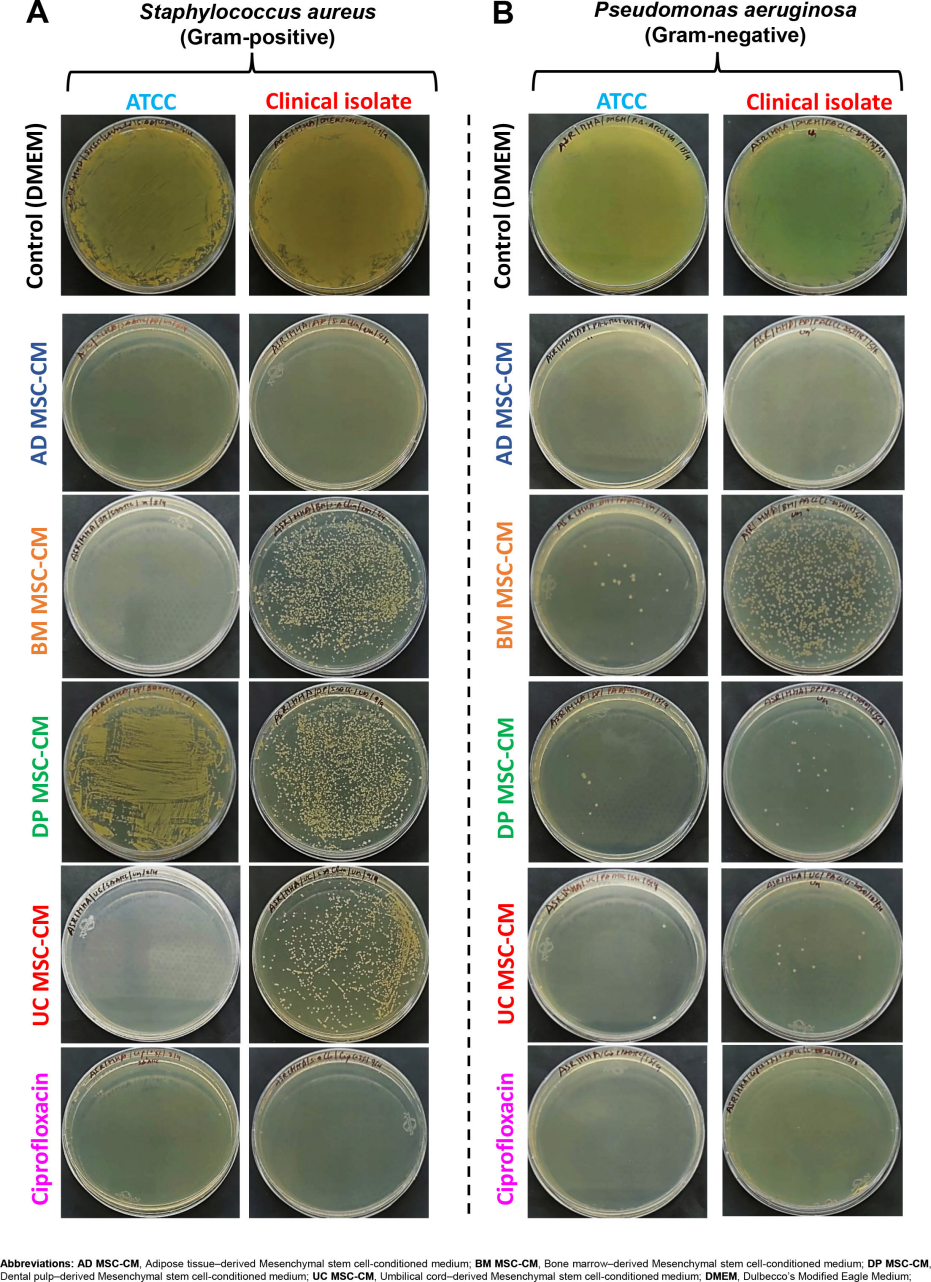
Effect of MSC-CM on the growth of *S. aureus* and *P. aeruginosa*. Colony-forming unit assay demonstrating the antibacterial activity of AD-, BM-, DP, and UC-MSC-CM was performed against (**A**) *S. aureus* ATCC (#29213) and clinical isolate (#L-1791/20) and (**B**) *P. aeruginosa* ATCC (#27853) and clinical isolate (#L-2050/18). Bacterial suspensions (6 × 10^8^ CFU/mL) were incubated with 500 µL of respective MSC-CM or DMEM (control) for 18 hours at 37°C. Representative images display agar plates for each treatment group (undiluted) following incubation for 24 hours at 37°C. Detailed quantitative analysis, including serial dilutions and graphical representations, is provided in Fig. S1.

MSC-CM demonstrated potent antibacterial activity against *S. aureus*, resulting in marked reductions in bacterial burden (Fig. 2A; Fig. S1a). When both the ATCC (#29213) and the clinical isolate (#L-1791/20) of *S. aureus* were treated with MSC-CM derived from AD-, BM-, DP-, or UC-MSCs, significant inhibition of bacterial growth was observed compared with the DMEM control (Fig. 2A).

For the ATCC (#29213) strain of *S. aureus*, the control group exhibited a bacterial load of 1.63 × 10^9^ CFU/mL. Treatment with AD-, BM-, and UC-derived MSC-CM reduced bacterial counts to below the limit of detection (LOD) across the dilutions tested, whereas DP-MSC-CM significantly reduced bacterial load to 3.46 × 10^7^ CFU/mL relative to the control (one-way ANOVA with Dunnett’s *post hoc* test, *P* < 0.0001) (Fig. S1a-i).

Similarly, for the clinical isolate of *S. aureus* (#L-1791/20), the control group showed 1.74 × 10^9^ CFU/mL. AD-MSC-CM reduced bacterial counts to below LOD, while BM-, DP-, and UC-MSC-CM significantly decreased CFU levels to 2.03 × 10^7^, 1.69 × 10^8^, and 9.0 × 10^5^ CFU/mL, respectively (*P* < 0.0001 vs control) (Fig. S1a-ii).

The LOD was defined based on the lowest dilution plated (10⁻⁶), and samples without detectable colonies were reported accordingly. Collectively, these findings highlight the robust antibacterial activity of MSC-CM, particularly of AD-derived MSC-CM, against both the reference strain and clinical isolates of *S. aureus*.

### MSC-CM inhibits the growth of the ATCC strain and the clinical isolate of *P. aeruginosa*

MSC-CM also exhibited strong antibacterial efficacy against *P. aeruginosa*, resulting in substantial reductions in bacterial burden (Fig. 2B; Fig. S1b). For the ATCC (#27853) strain, the control group demonstrated a bacterial load of 1.10 × 10^9^ CFU/mL. Treatment with MSC-CM derived from AD, BM, DP, and UC reduced bacterial counts below the LOD across all sources (one-way ANOVA with Dunnett’s *post hoc* test, *P* < 0.0001 vs control) (Fig. S1b-i).

In the clinical isolate (#L-2050/18), the control group showed 8.62 × 10^8^ CFU/mL, MSC-CM from AD, DP, and UC reduced bacterial counts to below LOD, while BM-MSC-CM significantly reduced bacterial burden to 1.98 × 10^7^ CFU/mL compared with the control (*P* < 0.0001) (Fig. S1b-ii). These results demonstrate consistent and pronounced antibacterial activity of MSC-CM against both reference and clinical *P. aeruginosa* isolates. The use of LOD-based reporting ensures accurate interpretation within the sensitivity limits of the CFU assay (Fig. S1b).

### MSC-CM demonstrates rapid and sustained antibacterial activity in time-course analysis

Time-kill analysis revealed robust bacterial growth in control cultures over 24 hours (Fig. S2). In contrast, MSC-CM treatment resulted in a rapid reduction in bacterial burden, with qualitative assessment of undiluted samples showing counts falling below the limit of detection as early as 2 hours under several conditions. This inhibitory effect was sustained through 24 hours, as confirmed by representative agar plate images at both the 2- and 24-hour intervals. Similar trends were observed for both ATCC and clinical isolates of *S. aureus* (Fig. S2a) and *P. aeruginosa* (Fig. S2b). Detailed quantitative time-course data, derived from aliquots collected at 2, 4, 6, 12, and 24 hours and plated at a 10⁻⁶ dilution to define the LOD, are provided in Fig. S2.

### MSC-CM contains antimicrobial peptides

**Fig 3:**
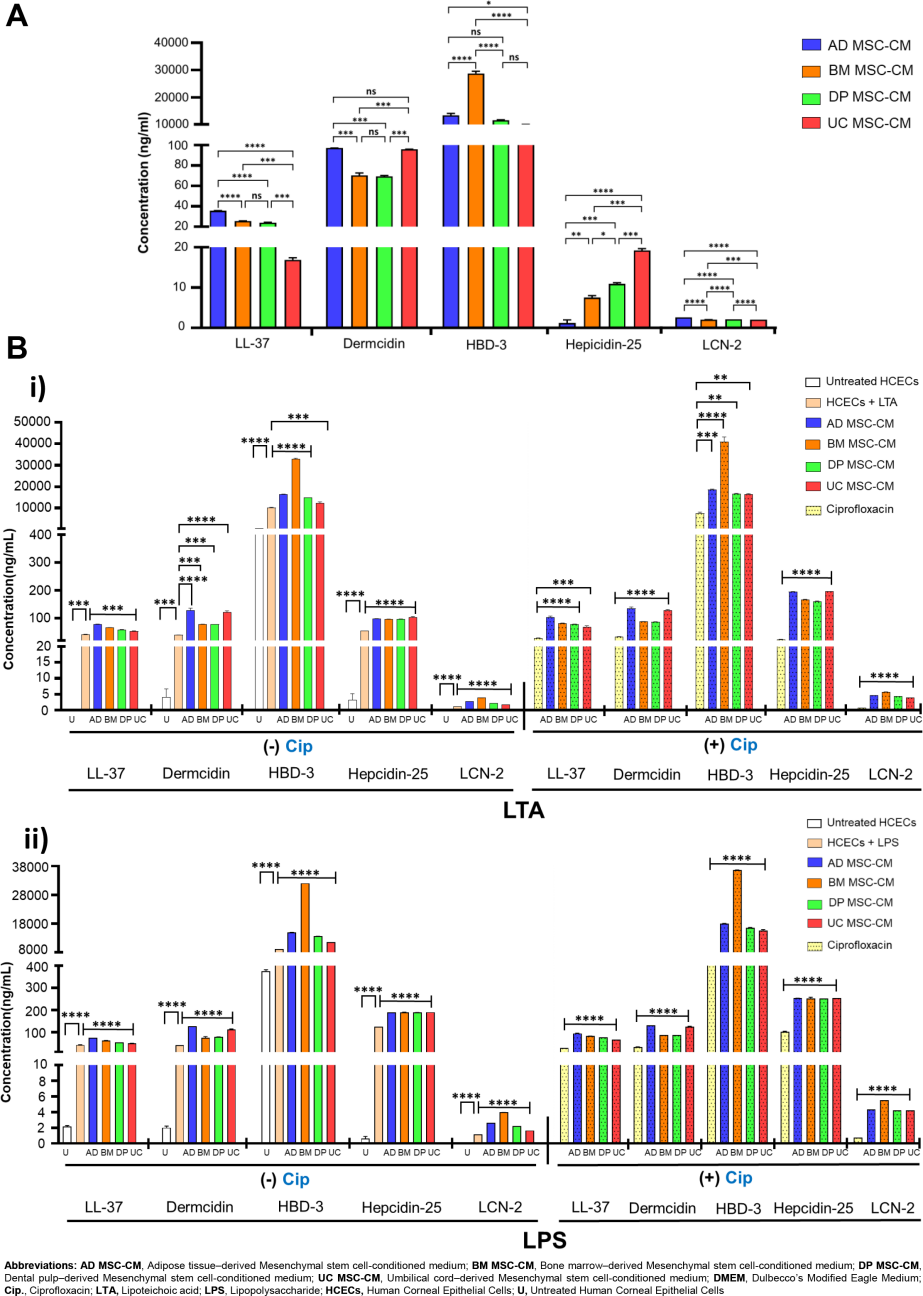
Expression of antimicrobial peptides in MSC-CM and their modulation upon induction with bacterial endotoxins. (**A**) Quantification of antimicrobial peptides (LL-37, Dermcidin, HBD-3, Hepcidin-25, and LCN-2) in MSC-CM derived from AD-, BM-, DP, and UC-MSCs. MSCs were cultured to 75% confluency in antibiotic-free DMEM supplemented with 10% FBS, followed by serum-free conditioning for 24 hours. AMP concentrations were measured by ELISA. Data are presented as mean ± SD; statistical comparisons among MSC-CM groups were performed using one-way ANOVA followed by Tukey’s multiple comparisons test. Significance levels are indicated as *P < 0.05; **P < 0.01; ***P < 0.001; ****P < 0.0001; and ns, not significant. (**B**) Modulation of AMP expression in HCECs following stimulation with bacterial endotoxins. HCECs were stimulated with 10 µg/mL lipoteichoic acid (i) or lipopolysaccharide (ii) for 24 hours, followed by treatment with MSC-CM for an additional 24 hours. Supernatants were collected and analyzed by ELISA for LL-37, Dermcidin, HBD-3, Hepcidin-25, and LCN-2. Comparisons were performed among untreated HCECs, stimulated control (HCECs + LTA/LPS), and MSC-CM-treated groups, and ciprofloxacin-treated groups using one-way ANOVA, followed by Dunnett’s multiple comparisons test. For stimulation analyses, the respective stimulated control (HCECs + LTA/LPS) served as the reference, while for evaluating therapeutic efficacy, ciprofloxacin-treated cells were designated as the reference group for comparison with MSC-CM-treated groups. Data are presented as mean ± SD. Significance levels are indicated as *P < 0.05; **P < 0.01; ***P < 0.001; ****P < 0.0001; ns, not significant.

The concentrations of AMPs (LL-37, Dermcidin, HBD-3, Hepcidin-25, and LCN-2) in AD-, BM-, DP-, and UC-derived MSC-CM were quantified by ELISA (Fig. 3A; Table 1). All five AMPs were detectable across MSC-CM derived from the different tissue sources.

**TABLE 1:** Baseline expression levels (ng/mL) of antimicrobial peptides in MSC-CM from four tissue sources^*a*^

AMPs	AD-MSC-CM	BM-MSC-CM	DP-MSC-CM	UC-MSC-CM
LL-37	35.61 ± 0.19	25.41 ± 0.22	23.85 ± 0.58	16.91 ± 0.48
Dermcidin	97.11 ± 0.18	70.24 ± 2.45	69.27 ± 0.85	95.83 ± 0.18
HBD-3	13,325.61 ± 0.80	28,762.77 ± 0.85	11,579.77 ± 0.14	9,970.07 ± 0.11
Hepcidin-25	1.22 ± 0.81	7.55 ± 0.51	10.96 ± 0.25	19.23 ± 0.47
LCN-2	2.037 ± 4.20	2.273 ± 4.45	1.886 ± 1.01	1.782 ± 4.95

^*a*^ All antimicrobial peptides were detectable at baseline across MSC-CM sources. Dermcidin showed significantly higher levels in AD- and UC-MSC-CM; HBD-3 was elevated in AD- and BM-MSC-CM; LL-37 was most abundant in AD-MSC-CM; Hepcidin-25 was markedly expressed in UC- and DP-MSC-CM; and LCN-2 was uniformly detected across all MSC-CM samples, with BM-MSC-CM exhibiting the highest baseline concentration. Tukey’s multiple comparisons test results for the five AMPs across MSC-CM sources are as follows: for LL-37, AD vs BM, DP, and UC comparisons were all significant (****P < 0.0001), while BM vs DP was not significant (*P* = 0.0598, ns), whereas BM vs UC (****P < 0.0001) and DP vs UC (***P = 0.0002) were significant. For Dermcidin, AD vs BM and AD vs DP were significant (***P = 0.0001), while AD vs UC (*P* = 0.7653, ns) and BM vs DP (*P* = 0.8759, ns) were not significant. BM vs UC (***P = 0.0002) and DP vs UC (***P = 0.0001) were significant. For HBD-3, AD vs BM, BM vs DP, and BM vs UC were highly significant (****P < 0.0001), AD vs UC was significant (*P = 0.0160), whereas AD vs DP (*P* = 0.1289, ns) and DP vs UC (*P* = 0.1597, ns) were not significant. For Hepcidin-25, all pairwise comparisons were significant: AD vs BM (**P < 0.01), AD vs DP (***P < 0.001), AD vs UC (****P < 0.0001), BM vs DP (*P < 0.05), BM vs UC (***P < 0.001), and DP vs UC (***P < 0.001). For LCN-2, AD vs BM, AD vs DP, AD vs UC, BM vs DP, and DP vs UC were highly significant (****P < 0.0001), while BM vs UC was also significant (***P < 0.001).

Comparative analysis among MSC-CM groups was performed using one-way ANOVA followed by Tukey’s multiple comparisons test. Dermcidin levels were significantly higher in AD- and UC-MSC-CM, while HBD-3 concentrations were the highest and significantly elevated in BM- and AD-MSC-CM. LL-37 was abundantly detected in all MSC-CM samples, with AD-MSC-CM exhibiting the highest concentration. HBD-3 demonstrated the highest overall abundance among the AMPs measured, particularly in BM-MSC-CM. Hepcidin-25 levels were notably elevated in UC-MSC-CM, while LCN-2 was detected in all MSC-CM groups, with significantly higher expression in BM-MSC-CM (Fig. 3A). Collectively, these data demonstrate that MSC-CM contains a diverse repertoire of AMPs, with source-dependent variations in the abundance of the tested AMPs.

Interestingly, among all the AMPs detected in different MSC-CM, HBD-3 was measured at the highest concentrations, followed by Dermcidin, LL-37, Hepcidin-25, and LCN-2 in a sequential manner (Fig. 3A; Table 1).

### MSC-CM upregulates the expression of AMPs in LTA-stimulated HCECs

To evaluate the immunomodulatory effect of MSC-CM under inflammation-mimicking conditions, HCECs were first stimulated with lipoteichoic acid to simulate gram-positive bacterial inflammation, followed by treatment with MSC-CM. Baseline AMP expression was detectable in untreated HCECs, constitutive of ocular surface innate immunity, as previously reported (34–36).

Comparisons were performed between LTA-stimulated cells (HCECs +LTA) and LTA-stimulated cells treated with MSC-CM (HCECs + LTA + MSC-CM) using one-way ANOVA followed by Dunnett’s multiple comparisons test (Fig. 3B-i). Treatment with ciprofloxacin [(+) Cip, represented through dotted bar graphs] was used as a control.

As shown in Table 2, which contains the quantitative values and statistical significance, MSC-CM treatment significantly enhanced the expression of LL-37 and Dermcidin compared with LTA-stimulated controls, with the highest induction observed following AD-MSC-CM treatment. HBD-3 was robustly upregulated, particularly in BM-MSC-CM-treated cells. Furthermore, Hepcidin-25 levels were significantly increased, with maximal expression observed following UC-MSC-CM treatment, while LCN-2 demonstrated a modest modulation. Notably, the administration of MSC-CM in combination with ciprofloxacin “(+) Cip” significantly enhanced AMP expression compared to ciprofloxacin alone (Table 3). In these combination groups, levels of LL-37 were the highest in the AD-MSC-CM + ciprofloxacin group, while Dermcidin expression was the most markedly elevated in the AD and UC combination groups. HBD-3 and LCN-2 exhibited the highest levels in the BM-MSC-CM + ciprofloxacin group, and Hepcidin-25 showed significant increases across all combination groups, particularly with AD and UC sources (Fig. 3B-i).

**TABLE 2:** AMP expression (ng/mL) in LTA-/LPS-stimulated HCECs treated with MSC-CM^*a*^

	LTA-stimulated HCECs					LPS-stimulated HCECs				
AMPs	Control	MSC-CM				Control	MSC-CM			
		AD	BM	DP	UC		AD	BM	DP	UC
LL-37	42.69 ± 0.54	78.62 ± 0.86 (***P < 0.001)	66.15 ± 1.03 (***P < 0.001)	59.18 ± 0.40 (***P < 0.001)	53.51 ± 1.46 (***P < 0.001)	42.21 ± 0.067	73.478 ± 0.158 (****P < 0.0001)	61.789 ± 0.260 (****P < 0.0001)	53.884 ± 0.071 (****P < 0.0001)	48.360 ± 1.111 (****P < 0.0001)
Dermcidin	40.05 ± 0.16	127.70 ± 8.43 (****P < 0.0001)	78.74 ± 0.20 (***P = 0.0004)	78.25 ± 0.16 (***P = 0.0004)	121.00 ± 5.35 (****P < 0.0001)	40.05 ± 0.10	125.707 ± 0.056 (****P < 0.0001)	75.052 ± 4.112 (****P < 0.0001)	77.673 ± 0.081 (****P < 0.0001)	111.69 ± 2.318 (****P < 0.0001)
HBD-3	10,094.59 ± 0.21	16,302.02 ± 0.16 (****P < 0.0001)	32,955.68 ± 0.32 (****P < 0.0001)	14,765.56 ± 0.02 (****P < 0.0001)	12,277.44 ± 0.54 (***P = 0.0009)	9,242.12 ± 0.007	14,983.460 ± 0.033 (****P < 0.0001)	32,061.755 ± 0.01 (****P < 0.0001)	13,632.9 ± 0.024 (****P < 0.0001)	11,601.20 ± 0.015 (****P < 0.0001)
Hepcidin-25	54.98 ± 0.95	98.24 ± 0.70 (****P < 0.0001)	95.99 ± 0.94 (****P < 0.0001)	96.48 ± 1.01 (****P < 0.0001)	103.11 ± 2.96 (****P < 0.0001)	124.89 ± 0.13	189.210 ± 0.166 (****P < 0.0001)	188.935 ± 0.776 (****P < 0.0001)		189.95 ± 0.111 (****P < 0.0001)
									188.543 ± 0.333 (****P < 0.0001)	
LCN-2	1.18 ± 3.48	2.83 ± 5.16 (****P < 0.0001)	3.85 ± 3.69 (****P < 0.0001)	2.24 ± 6.61 (****P < 0.0001)	1.71 ± 2.92 (****P < 0.0001)	1.15 ± 0.86	2.660 ± 4.924 (****P < 0.0001)	3.970 ± 1.939 (****P < 0.0001)	2.215 ± 5.476 (****P < 0.0001)	1.679 ± 1.933 (****P < 0.0001)

^*a*^ MSC-CM derived from adipose, bone marrow, dental pulp, and umbilical cord tissues significantly enhanced antimicrobial peptide expression in LTA- and LPS-stimulated human corneal epithelial cells. In LTA-stimulated HCECs, LL-37 levels were the highest in the AD-MSC-CM (78.62 ± 0.86 ng/mL, ***), while Dermcidin was the most elevated with AD- and UC-MSC-CM (127.70 ± 8.43 and 121.00 ± 5.35 ng/mL, respectively). HBD-3 exhibited a higher level in BM-MSC-CM (32,955.68 ± 0.32 ng/mL, ****), with a notable increase also seen in the AD group. Hepcidin-25 was upregulated across all MSC-CM groups, with UC- and AD-MSC-CM showing the highest levels (103.11 ± 2.96 and 98.24 ± 0.70 ng/mL, respectively). LCN-2 expression was significantly enhanced in all groups, particularly with BM-MSC-CM (3.85 ± 3.69 ng/mL, ****) followed by AD. In LPS-stimulated HCECs, LL-37 remained the highest in the AD-MSC-CM group (73.478 ± 0.158 ng/mL, ****), while Dermcidin also peaked with AD-MSC-CM (125.707 ± 0.056 ng/mL, ****), followed by UC (111.69 ± 2.318 ng/mL, ****). HBD-3 again showed a maximal level with BM-MSC-CM (32,061.755 ± 0.016 ng/mL, ****), followed by the AD group. Hepcidin-25 levels were uniformly elevated, reaching the highest values with UC- and AD-MSC-CM (189.95 ± 0.111 and 189.210 ± 0.166 ng/mL, respectively). Finally, LCN-2 was strongly upregulated, with the BM-MSC-CM group again showing the highest levels (3.970 ± 1.939 ng/mL, ****) followed by AD. Data are shown as mean ± SD (*P < 0.05; **P < 0.01; ***P < 0.001; ****P < 0.0001; and ns, not significant).

**TABLE 3:** AMP expression (ng/mL) in LTA-/LPS-stimulated HCECs treated with MSC-CM in combination with ciprofloxacin^*a*^

	LTA-stimulated HCECs					LPS-stimulated HCECs				
AMPs	Ciprofloxacin	MSC-CM + ciprofloxacin				Ciprofloxacin	MSC-CM + ciprofloxacin			
		AD	BM	DP	UC		AD	BM	DP	UC
LL-37	28.68 ± 1.20	103.43 ± 4.23 (****P < 0.0001)	82.14 ± 0.66 (****P < 0.0001)	77.73 ± 1.78 (****P < 0.0001)	69.12 ± 3.86 (***P = 0.0001)	27.85 ± 0.09	94.01 ± 0.93 (****P < 0.0001)	81.73 ± 0.08 (****P < 0.0001)	75.53± 0.120 (****P < 0.0001)	66.47 ± 0.038 (****P < 0.0001)
Dermcidin	33.50 ± 0.74	135.16 ± 5.20 (****P < 0.0001)	87.86 ± 0.90 (****P < 0.0001)	86.95 ± 0.47 (****P < 0.0001)	128.00 ± 3.25 (****P < 0.0001)	32.71 ± 0.49	130.55 ± 0.11 (****P < 0.0001)	87.25 ± 0.12 (****P < 0.0001)	85.98 ± 0.04 (****P < 0.0001)	124.09 ± 2.26 (****P < 0.0001)
HBD-3	7,507.81 ± 0.57	18,555.95 ± 0.31 (***P = 0.0005)	40,867.06 ± 2.35 (****P < 0.0001)	16,629.08 ± 0.24 (**P = 0.0013)	16,505.26 ± 0.20 (**P = 0.0014)	7,277.38 ± 0.03	18,033.63 ± 0.05 (****P < 0.0001)	36,618.77 ± 0.06 (****P < 0.0001)	16,634.85 ± 0.04 (****P < 0.0001)	15,676.18 ± 0.30 (****P < 0.0001)
Hepcidin-25	24.49 ± 0.22	195.18 ± 0.48 (****P < 0.0001)	167.22 ± 1.01 (****P < 0.0001)	158.90 ± 3.30 (****P < 0.0001)	195.87± 0.37 (****P < 0.0001)	102.04 ± 1.59	252.90 ± 0.36 (****P < 0.0001)	252.24 ± 4.62 (****P < 0.0001)	252.19 ± 0.09 (****P < 0.0001)	253.46 ± 0.04 (****P < 0.0001)
LCN-2	0.785 ± 6.46	4.68 ± 5.64 (****P < 0.0001)	5.74 ± 8.68 (****P < 0.0001)	4.34 ± 2.31 (****P < 0.0001)	3.87 ± 1.85 (****P < 0.0001)	0.783 ± 4.04	4.32 ± 4.60 (****P < 0.0001)	5.55 ± 6.35 (****P < 0.0001)	4.23 ± 2.19 (****P < 0.0001)	3.82 ± 3.64 + (****P < 0.0001)

^*a*^ MSC-CM derived from adipose, bone marrow, dental pulp, and umbilical cord tissues significantly enhanced antimicrobial peptide expression when administered in combination with ciprofloxacin in LTA- and LPS-stimulated human corneal epithelial cells, compared with ciprofloxacin alone. In LTA-stimulated HCECs, LL-37 levels were the highest in the AD-MSC-CM + ciprofloxacin group (103.43 ± 4.23 ng/mL, ****), followed by BM-, DP-, and UC-MSC-CM combination groups, all showing significant increases compared with ciprofloxacin alone. Dermcidin expression was markedly elevated in AD- and UC-MSC-CM + ciprofloxacin groups (135.16 ± 5.20 and 128.00 ± 3.25 ng/mL, respectively, ****). HBD-3 exhibited the highest levels in the BM-MSC-CM + ciprofloxacin group (40,867.06 ± 2.35 ng/mL, ****), with a significant upregulation also observed in AD-, DP-, and UC-MSC-CM combination groups. Hepcidin-25 was significantly increased across all MSC-CM + ciprofloxacin groups, with the highest expression observed in AD- and UC-MSC-CM groups (195.18 ± 0.48 and 195.87 ± 0.37 ng/mL, respectively, ****). LCN-2 expression was also significantly elevated, particularly in the BM-MSC-CM + ciprofloxacin group (5.74 ± 8.6 ng/mL, ****), followed by AD-, DP-, and UC-MSC-CM combination groups. In LPS-stimulated HCECs, LL-37 levels remained the highest in the AD-MSC-CM + ciprofloxacin group (94.01 ± 0.93 ng/mL, ****), followed by BM-, DP-, and UC-MSC-CM combination groups. Dermcidin expression was significantly elevated in AD- and UC-MSC-CM combination groups (130.55 ± 0.11 and 109.69 ± 2.26 ng/mL, respectively, ****). HBD-3 again showed a maximal expression in the BM-MSC-CM + ciprofloxacin group (36,618.77 ± 0.06 ng/mL, ****), followed by the AD-, DP-, and UC-MSC-CM groups. Hepcidin-25 levels were uniformly elevated across all MSC-CM + ciprofloxacin groups, with the highest expression observed in UC- and AD-MSC-CM (253.46 ± 0.04 and 252.90 ± 0.36 ng/mL, respectively, ****). Finally, LCN-2 was significantly increased, with the BM-MSC-CM + ciprofloxacin group showing the highest levels (5.55 ± 6.35 ng/mL, ****), followed by AD-, DP-, and UC-MSC-CM combination groups. Data are shown as mean ± SD (*P < 0.05; **P < 0.01; ***P < 0.001; ****P < 0.0001; and ns, not significant).

### MSC-CM upregulates the expression of AMPs in LPS-stimulated HCECs

To model gram-negative bacterial inflammation, HCECs were stimulated with LPS followed by MSC-CM treatment. Following the same statistical approach as for LTA groups, analysis was performed using one-way ANOVA with Dunnett’s *post hoc* testing, comparing LPS-stimulated control cells to MSC-CM-treated groups (Fig. 3B-ii; Table 2).

The results with outcomes of statistical analyses are summarized in Table 2 and demonstrate that treatment with MSC-CM significantly increased LL-37 and Dermcidin levels across all MSC sources, with AD-MSC-CM again yielding the most significant elevation. HBD-3 expression was markedly elevated in BM-MSC-CM-treated cells. In the case of Hepcidin-25 and LCN-2, significant increases were observed, with the strongest responses attributed to UC-MSC-CM and BM-MSC-CM, respectively. Similar to the LTA group, the combination of MSC-CM and ciprofloxacin “(+) Cip” resulted in a greater upregulation of AMPs compared to antibiotic treatment alone (Table 3). In LPS-stimulated HCECs, LL-37 and Dermcidin levels remained the highest in the AD and UC combination groups. HBD-3 and LCN-2 again showed maximal expression in the BM-MSC-CM + ciprofloxacin group. Hepcidin-25 levels were uniformly elevated across all combination groups, reaching higher values with UC- and AD-MSC-CM.

Together, these results demonstrate that MSC-CM amplifies antimicrobial peptide responses in corneal epithelial cells under both gram-positive and gram-negative inflammatory conditions.

### MSC-CM downregulates the expression of pro-inflammatory cytokines in LTA-/LPS-stimulated HCECs

**Fig 4:**
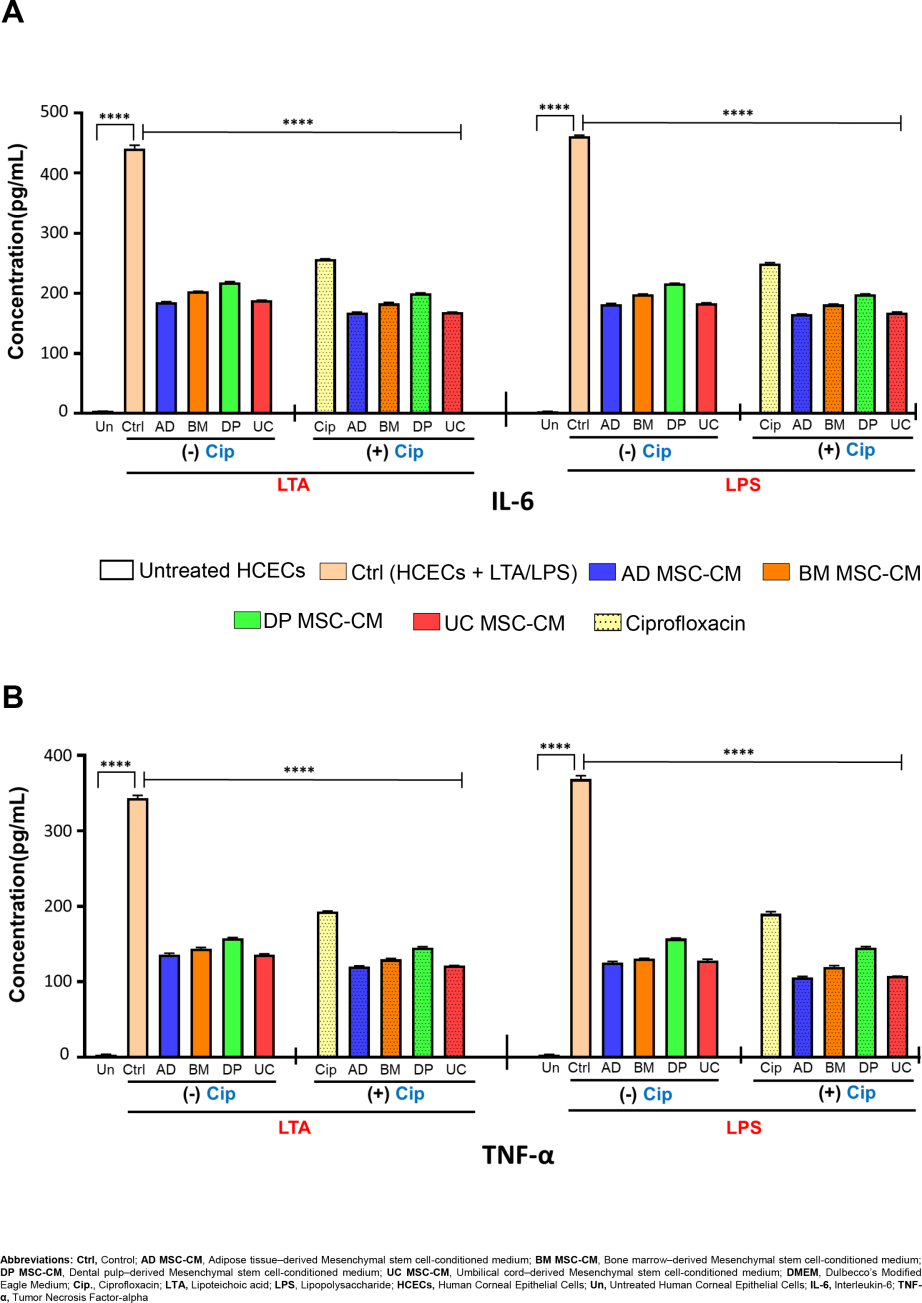
Modulation of pro-inflammatory cytokine expression in LTA-/LPS-stimulated HCECs following treatment with MSC-CM. ELISA quantification of (**A**) IL-6 and (**B**) TNF-α levels in HCECs stimulated with 10 µg/mL LTA or LPS for 24 hours, followed by treatment with AD-, BM-, DP-, and UC-MSC-CM for an additional 24 hours. Culture supernatants were collected and analyzed by ELISA. Comparisons were performed among untreated (Un), stimulated control (Ctrl) (HCECs + LTA/LPS), MSC-CM-treated, and ciprofloxacin-treated groups using one-way ANOVA followed by Dunnett’s multiple comparisons test; the respective stimulated control served as the reference group. Data are presented as mean ± SD. Significance levels are indicated as *P < 0.05; **P < 0.01; ***P < 0.001; and ****P < 0.0001.

HCECs stimulated with LTA or LPS (“Ctrl”) exhibited significantly increased expression of pro-inflammatory cytokines IL-6 and TNF-α compared with unstimulated cells (“Un”), as shown in Fig. 4. Subsequent treatment with MSC-CM resulted in significant downregulation of IL-6 and TNF-α levels relative to the respective stimulated controls (Fig. 4A and B; Table 4). Statistical comparisons were performed using one-way ANOVA followed by Dunnett’s multiple comparisons test, with stimulated control cells (“Ctrl”) serving as the reference group. The magnitude of cytokine reduction varied depending on the MSC source, but overall, AD-MSC-CM demonstrated the most consistent and significant anti-inflammatory effects under both LTA- and LPS-induced conditions (Fig. 4). This anti-inflammatory activity was further enhanced when MSC-CM was administered in combination with ciprofloxacin [“(+) Cip”], resulting in significantly lower cytokine concentrations (Table 4). In these combination groups, AD-MSC-CM plus ciprofloxacin consistently achieved the maximal reduction in cytokine expression across both LTA and LPS models. These findings indicate that MSC-CM from various sources, particularly adipose tissue, effectively mitigates the inflammatory response. Collectively, these results support the dual antibacterial and immunomodulatory properties of MSC-CM in the context of bacterial keratitis *in vitro*.

**TABLE 4:** Effect of MSC-CM treatment on the expression of pro-inflammatory cytokines in LTA-/LPS-stimulated HCECs^*a*^

(−) Cip	LTA-stimulated HCECs					LPS-stimulated HCECs				
Cytokines	Control	MSC-CM				Control	MSC-CM			
		AD	BM	DP	UC		AD	BM	DP	UC
IL-6	440.88 ± 5.31	184.98± 1.28 (****P < 0.0001)	203.18 ± 0.24 (****P < 0.0001)	218.25 ± 1.42 (****P < 0.0001)	188.21 ± 0.23 (****P < 0.0001)	461.62 ± 1.29	181.95 ± 1.15 (****P < 0.0001)	198.52 ± 0.48 (****P < 0.0001)	215.98 ± 0.51 (****P < 0.0001)	183.83 ± 0.58 (****P < 0.0001)
TNF- α	343.42 ± 3.69	135.99 ± 1.99 (****P < 0.0001)	143.78 ± 1.85 (****P < 0.0001)	157.47 ± 1.25 (****P < 0.0001)	136.18± 0.81 (****P < 0.0001)	368.74 ± 4.52	125.22 ± 1.94 (****P < 0.0001)	130.76 ± 0.53 (****P < 0.0001)	157.33 ± 0.86 (****P < 0.0001 )	128.10 ± 1.78 (****P < 0.0001)

(+) Cip	LTA-stimulated HCECs					LPS-stimulated HCECs				
Cytokines	Ciprofloxacin	MSC-CM + ciprofloxacin				Ciprofloxacin	MSC-CM + ciprofloxacin			
		AD	BM	DP	UC		AD	BM	DP	UC
IL-6	256.59 ± 0.88	167.64 ± 1.21 (****P < 0.0001)	183.26 ± 1.62 (****P < 0.0001)	199.89 ± 0.97 (****P < 0.0001)	168.58 ± 0.33 (****P < 0.0001)	249.67 ± 1.29	165.53 ± 0.22 (****P < 0.0001)	181.46 ± 0.69 (****P < 0.0001)	198.09 ± 1.09 (****P < 0.0001)	168.19 ± 0.88 (****P < 0.0001)
TNF-α	193.22 ± 0.74	120.24 ± 0.87 (****P < 0.0001)	129.74 ± 1.25 (****P < 0.0001)	145.49 ± 1.11 (****P < 0.0001)	121.29 ± 0.43 (****P < 0.0001)	190.23 ± 2.85	105.92 ± 1.34 (****P < 0.0001)	119.63 ± 1.91 (****P < 0.0001)	145.29 ± 1.39 (****P < 0.0001)	107.47 ± 0.16 (****P < 0.0001)

^*a*^ MSC-CM significantly suppressed pro-inflammatory cytokine expression in both LTA- and LPS-stimulated HCECs compared to control (****P < 0.0001), with AD-MSC-CM demonstrating the most potent inhibitory effect on IL-6 and TNF- α. Specifically, in LTA-stimulated cells, AD-MSC-CM reduced IL-6 to 184.98 ± 1.28 pg/mL and TNF-α to 135.99 ± 1.99 pg/mL, while in LPS-stimulated cells, levels were lowered to 181.95 ± 1.15 and 125.22 ± 1.94 pg/mL, respectively. This anti-inflammatory activity was further enhanced when MSC-CM was combined with ciprofloxacin, resulting in significantly lower cytokine concentration compared to antibiotic treatment alone (****P < 0.0001). In these combination groups, AD-MSC-CM plus ciprofloxacin consistently achieved the maximal reduction in cytokine expression across LTA and LPS models, with IL-6 falling to 165.53 ± 0.22 pg/mL and TNF-α to 105.92 ± 1.34 pg/mL in the LPS-stimulated group. These findings indicate that MSC-CM from various sources, particularly adipose tissue, effectively mitigates the inflammatory response and gives a protective effect in combination with conventional antibiotics.

### AD MSC-CM improves corneal transparency in *ex vivo* infected human corneas

**Fig 5:**
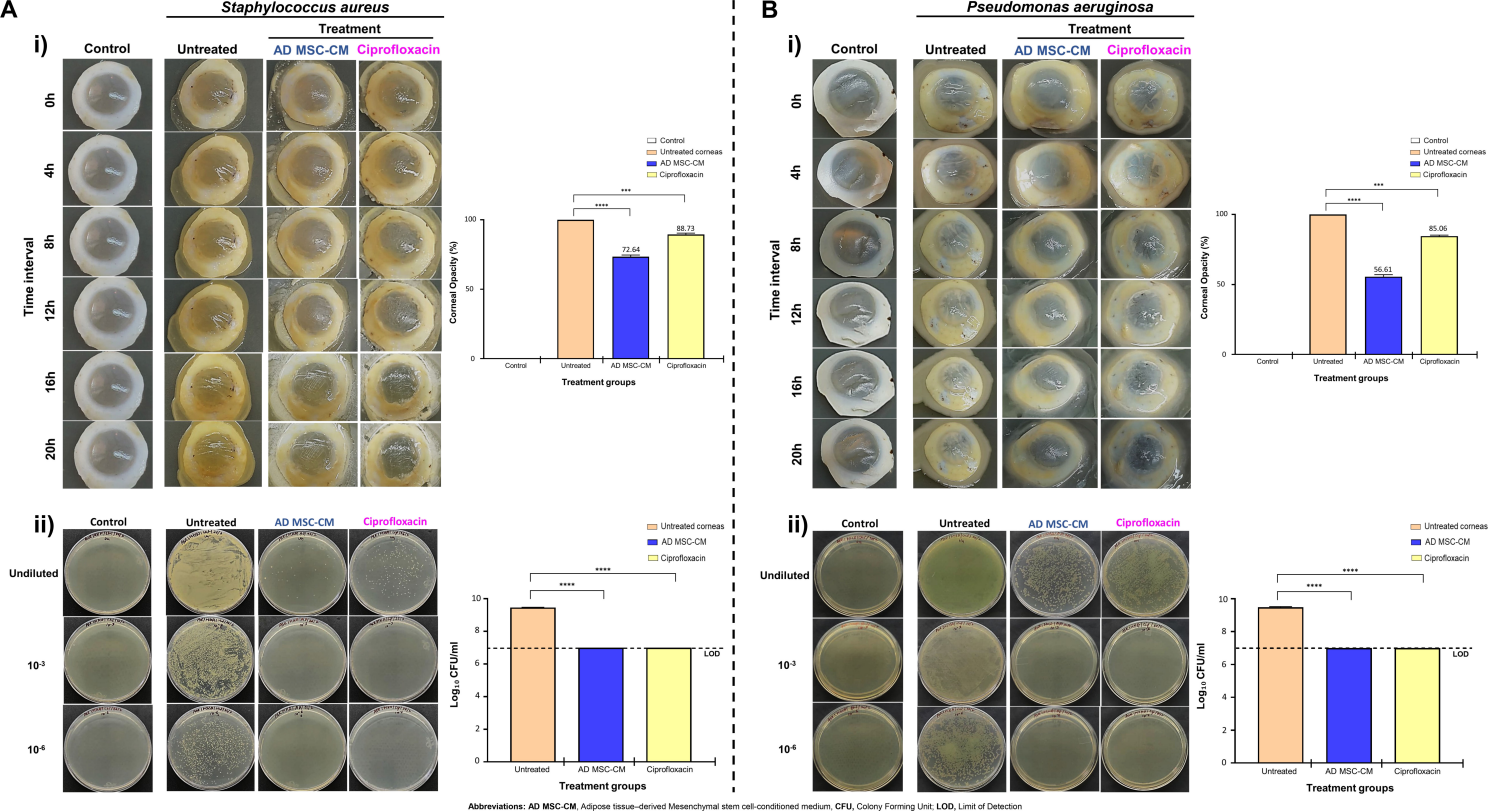
AD-MSC-CM improves corneal transparency and reduces bacterial burden in *ex vivo* infected human corneas. Human donor corneas were sterilized, supported in MHA molds, and centrally injured via 26-G needle before being infected with (**A**) *S. aureus* or (**B**) *P. aeruginosa*. After 24-hour infection establishment, corneas were treated topically with ciprofloxacin or adipose-derived mesenchymal stem cell-conditioned medium. (i) Corneal opacity analysis showed complete loss of transparency in untreated infected corneas (100% opacity). In the *S. aureus* model, corneal opacity was reduced to 88.73% with ciprofloxacin and 72.64% with AD-MSC-CM treatment, whereas in the *P. aeruginosa* model, corneal opacity was reduced to 85.06% and 56.61%, respectively. (ii) Bacterial burden, quantified by CFU assay of homogenized corneal buttons, demonstrated robust bacterial proliferation in untreated corneas (9.46 log_10_ CFU/mL for *S. aureus* and 9.48 log_10_ CFU/mL for *P. aeruginosa*), while both ciprofloxacin and AD-MSC-CM treatments reduced bacterial load to the limit of detection (7.00 log_10_ CFU/mL). Data are presented as mean ± SD. Statistical analysis was performed using one-way ANOVA followed by Dunnett’s multiple comparisons test (*P < 0.05; **P < 0.01; ***P < 0.001; and ****P < 0.0001).

Analysis of corneal opacity revealed a substantial loss of transparency in untreated infected corneas, which were normalized to 100% opacity for comparative analysis. In an *ex vivo* infection model utilizing clinical isolates to better correlate experimental findings with therapeutic efficacy, corneal opacity was assessed at the 20-hour endpoint. For *S. aureus* infection (Fig. 5A), ciprofloxacin treatment reduced opacity to 88.73%, while AD-MSC-CM treatment reduced opacity to 72.64% relative to untreated infected corneas. A similar trend was observed in the *P. aeruginosa* clinical isolate infection model (Fig. 5B); ciprofloxacin treatment improved corneal clarity, reducing opacity to 85.06% relative to untreated controls. Notably, AD-MSC-CM treatment resulted in greater improvement in corneal transparency, reducing opacity to 56.61% compared to untreated infected corneas. Together, these findings indicate that AD-MSC-CM treatment is associated with improved preservation of corneal clarity following bacterial infection (Fig. 5).

### AD-MSC-CM significantly reduces bacterial burden in *ex vivo* human corneas

An *ex vivo* human cornea infection model was established to assess the antibacterial efficacy of AD-MSC-CM against *S. aureus* and *P. aeruginosa*. Following 24 hours of infection establishment, untreated infected corneas exhibited a robust bacterial burden of 9.46 and 9.48 log_10_ CFU/mL for clinical isolates of *S. aureus* (Fig. 5A-ii) and *P. aeruginosa* (Fig. 5B-ii), confirming robust bacterial proliferation within corneal tissue. Treatment with ciprofloxacin reduced bacterial burden to the limit of detection (7.00 log_10_ CFU/mL) in both infection models. Similarly, AD-MSC-CM treatment reduced the bacterial burden to the limit of detection, representing an approximate 2.46-log reduction for the tested clinical isolates of *S. aureus* and a 2.48-log reduction for *P. aeruginosa* compared with untreated controls. Both treatments showed statistically significant reductions in bacterial load compared to untreated infected corneas (****P < 0.0001), supporting the antibacterial efficacy of AD-MSC-CM in an *ex vivo* human corneal infection model (Fig. 5A and B).

### MSC-CM effectively disrupts mature biofilms of *S. aureus* and *P. aeruginosa*

MSC-CM demonstrated potent inhibitory effects against pre-established biofilms of both *S. aureus* and *P. aeruginosa* (Fig. S3). In *S. aureus* ATCC (#29213), MSC-CM derived from AD-, BM-, DP-, and UC-MSCs resulted in 14.46%, 18.75%, 28.41%, and 16.62% biofilm growth, respectively, compared with untreated controls (100%), whereas ciprofloxacin showed maximal inhibition with only 1.52% biofilm growth. Dunnett’s multiple comparisons test (relative to ciprofloxacin) indicated that MSC-CM groups showed slightly lower but significant antibiofilm activity (****P < 0.0001). Similarly, in the clinical isolate of *S. aureus* (#L-1791/20), MSC-CM derived from AD-, BM-, DP-, and UC-MSCs showed 3.65%, 15.78%, 7.43%, and 7.82% growth, comparable to ciprofloxacin (6.54% growth), with AD-, DP-, and UC-MSC-CM showing no significant difference relative to ciprofloxacin (*P* > 0.05).

In *P. aeruginosa* ATCC (#27953), MSC-CM derived from AD-, BM-, DP-, and UC-MSCs resulted in 13.50%, 17.95%, 30.21%, and 19.35% growth, while ciprofloxacin achieved 8.25% growth, with MSC-CM showing significant but slightly lower activity (****P < 0.0001). In the clinical isolate of *P. aeruginosa* (#L-2050/18), MSC-CM derived from AD-, BM-, DP-, and UC-MSCs showed 3.46%, 20.36%, 6.54%, and 4.46% growth, which was comparable to ciprofloxacin (7.60% growth), with AD-, DP-, and UC-MSC-CM showing no significant difference (*P* > 0.05). Overall, these findings demonstrate that MSC-CM effectively inhibits pre-established biofilms of both *S. aureus* and *P. aeruginosa* ocular pathogens (Fig. S3a and b).

## DISCUSSION

This study provides a comprehensive insight into the antibacterial and immunomodulatory effects of MSC-CM against ocular isolates of *S. aureus* and *P. aeruginosa*, two primary pathogens implicated in bacterial keratitis. MSC-CM exhibited notable antibacterial effects against both *S. aureus* and *P. aeruginosa*, as evidenced by clear inhibition zones and distinct morphological changes under SEM (Fig. 1A and B). Untreated bacterial cells of both species showed normal morphology with smooth, intact surfaces, whereas MSC-CM-treated cells displayed clear deformation and surface irregularities (Fig. 1B). In *P. aeruginosa*, elongation and distortion of cells were particularly evident following treatment with MSC-CM, consistent with changes reported in ciprofloxacin-treated bacteria, thus supporting the antibacterial property of MSC-CM (37). The CFU assays demonstrated that MSC-CM from different sources significantly inhibited bacterial growth in both ATCC and clinical strains of these pathogens. Notably, adipose-derived MSC-CM showed the highest antibacterial activity among all MSC-CM sources tested in this study. This aligns with the recent findings where AD-MSC-CM was used against pathogens of urinary tract infections and skin wounds, respectively (14, 30). Additionally, AD-MSC-CM effectively inhibited clinical methicillin-resistant *Staphylococcus aureus* strains, further highlighting its antibacterial action (38). These results collectively underscore the broad-spectrum and higher antibacterial potential of AD MSC-CM compared with the BM-, DP-, and UC-MSC-CM, which in this study was also evident through the highest concentrations of HBD-3 and Dermcidin in AD-MSC-CM. MSC-CM effectively inhibits pre-established biofilms of *S. aureus* and *P. aeruginosa*, including both ATCC and clinical isolates (Fig. S3a and b). AD-, BM-, DP-, and UC-MSC-CM showed antibiofilm activity comparable to ciprofloxacin. The potent antibiofilm effect of ciprofloxacin observed in this study is consistent with previous reports showing its superior activity against *S. aureus* and *P. aeruginosa* biofilms and its ability to achieve >90% reduction in biofilm viability by penetrating the extracellular matrix (39, 40). These findings indicate that MSC-CM demonstrates measurable antibiofilm activity under the tested experimental conditions and can reduce pre-established biofilm burden.

Importantly, the antibacterial efficacy of MSC-CM was further validated in a clinically relevant *ex vivo* human corneal infection model (Fig. 5A and B). Untreated infected corneas exhibited extensive bacterial proliferation, whereas treatment with AD-MSC-CM significantly reduced bacterial burden to the limit of detection, comparable to ciprofloxacin treatment. In addition to bacterial clearance, AD-MSC-CM demonstrated improved preservation of corneal transparency compared with untreated controls and showed reduced infection-associated corneal opacity (Fig. 5A-i and B-i). This observation is particularly relevant, as preservation of corneal clarity is essential for maintaining visual function.

In line with the reports suggesting involvement of MSC-secretome in corneal wound healing, and their tissue-dependent variations in ocular surface diseases, these antibacterial effects are likely mediated by soluble antimicrobial peptides secreted by MSCs (41–47). We identified the presence and differential expression of LL-37, HBD-3, Dermcidin, Hepcidin-25, and LCN-2 across different MSC-CM, using ELISA. Notably, Dermcidin showed significantly higher levels in AD- and UC-MSC-CM, and HBD-3 showed significantly higher levels in BM- and AD-MSC-CM. These findings suggest that tissue origin plays a crucial role in determining the immunomodulatory and antimicrobial composition of MSC-CM, which may influence its therapeutic efficacy. These further underscore the hypothesis that synergistic action of different AMPs, particularly HBD-3 and Dermcidin in this study, contributes to their antibacterial effects. The higher levels of HBD-3 and Dermcidin, both known for their potent bactericidal activity, could explain the superior antibacterial performance observed in AD- and UC-derived MSC-CM (18, 48). The observed trend of higher antibacterial efficacy of MSC-CM against *P. aeruginosa* compared to *S. aureus* (Fig. 2) likely relates to the structural differences in bacterial cell envelopes. The outer membrane of gram-negative *P. aeruginosa* facilitates initial electrostatic interactions with cationic AMPs identified in the MSC-CM, such as HBD-3. Furthermore, the thinner peptidoglycan layer of *P. aeruginosa* may be more susceptible to the membrane-disrupting effects of these peptides compared to the dense, highly cross-linked peptidoglycan barrier of gram-positive *S. aureus*.

Importantly, to the best of our knowledge, this is the first study to report the presence of Dermcidin and HBD-3 in AD-, DP-, and UC-MSC-CM; Hepcidin-25 in AD- and DP-MSC-CM; and LCN-2 in DP-MSC-CM. These findings introduce novel insights into the AMP repertoire of MSC-CM from distinct sources and their contributions to host defense in the ocular microenvironment. These AMPs contribute to the direct antibacterial effects of MSC-CM, as evidenced by CFU reduction in tested isolates of *S. aureus* and *P. aeruginosa* (Fig. 2A and B).

Additionally, the immunomodulatory potential of MSC-CM was evaluated in HCECs stimulated with bacterial endotoxins: LTA and LPS, which are major etiologic components of gram-positive and gram-negative bacteria, respectively (49, 50). In both LTA- and LPS-stimulated conditions, we observed the enhanced AMP expression patterns following treatment with MSC-CM, confirming the consistency and robustness of MSC-CM immunomodulatory effects across different bacterial triggers. Additionally, MSC-CM further exhibited a significant immunomodulatory property by suppressing the production of key proinflammatory cytokines IL-6 and TNF-α in stimulated (LTA/LPS) HCECs. Arguably, being an *in vitro* study*,* the current work could have involved co-culture studies of HCECs with live bacteria (*S. aureus* and *P. aeruginosa*); however, maintaining viable HCECs with increasingly multiplying bacteria poses substantial technical challenges due to rapid bacterial overgrowth and cytotoxicity. To overcome this, we employed LTA and LPS stimulation models in related experiments to simulate infection-associated inflammatory conditions.

The dual benefits (antibacterial activity and immunomodulation) offered by MSC-CM in this study, in conjunction with our previous report, can be used to enhance the therapeutic efficacy in combination with antibiotics (19). Thus, MSC-CM could help overcome limitations by restoring or boosting innate defenses—an essential component in clearing bacterial keratitis. Moreover, MSC-CM, as a cell-free product, circumvents several challenges associated with live stem cell therapy, including immune rejection, ethical concerns, and complex regulatory hurdles.

Despite these encouraging results, certain limitations of this study must be acknowledged. While the clinical *S. aureus* isolate showed a comparatively smaller ZOI for ciprofloxacin, it is noteworthy that AD-MSC-CM produced a significantly larger ZOI than the antibiotic alone, suggesting potent activity even against isolates with reduced fluoroquinolone sensitivity. Our quantitative CFU and time-kill assays confirmed this bactericidal efficacy, which was further validated in an *ex vivo* human cornea infection model. In this *ex vivo* human cornea infection model, AD MSC-CM treatment reduced bacterial burden of both clinical isolates of *S. aureus* and *P. aeruginosa* to the limit of detection, achieving a significant 2.5-log reduction compared to the untreated controls (*P* < 0.0001; Fig. 5A and B). While these findings support the clinical potential of MSC-CM, future studies with a larger number of clinical isolates, specifically with multi-drug-resistant strains in combination with *in vivo* studies, are warranted to fully validate the clinical applicability of MSC-CM for the treatment of infectious keratitis, particularly in the context of increasing AMR. Though the studied isolates are clinically significant for ocular bacterial infections, we acknowledge that they may not encompass the full genetic and phenotypic diversity of ocular pathogens. Expanding the spectrum of tested isolates in future work will further substantiate the broad-spectrum antibacterial potential of MSC-CM. Additionally, *in vivo* validation using an established keratitis model is planned as part of our future research pipeline. Such studies will provide deeper mechanistic insights and strengthen translational relevance by evaluating the therapeutic efficacy of MSC-CM in a physiologically relevant context.

In summary, this study presents compelling evidence for the antibacterial and immunomodulatory potential of MSC-CM derived from human adipose tissue, bone marrow, dental pulp, and umbilical cord, against ocular bacterial pathogens. Notably, MSC-CM exhibited significant inhibitory effects against planktonic growth and pre-established biofilms of *Staphylococcus aureus* and *Pseudomonas aeruginosa*, and its antibacterial efficacy was further supported in a clinically relevant *ex vivo* human cornea infection model, where AD-MSC-CM markedly reduced bacterial burden and was associated with improved preservation of corneal transparency. The observed antibacterial activity is likely mediated by a mixture of soluble factors secreted by MSCs, including AMPs such as Dermcidin, HBD-3, LL-37, Hepcidin-25, and LCN-2, which are known to disrupt bacterial viability and modulate host defense mechanisms. Importantly, differences in antibacterial efficacy among MSC-CM derived from different tissue sources were associated with distinct AMP profiles, suggesting that the tissue origin of MSCs influences their therapeutic potential. The higher performance of specific MSC-CM (e.g., AD-MSCs in this case) may reflect a more robust innate antimicrobial peptide repertoire, the subject of further investigation, pointing toward the relevance of source selection in optimizing MSC-CM-based therapies. These findings, for the first time to the best of our knowledge, underscore the therapeutic potential of MSC-CM for bacterial keratitis.

## Data Availability

The data supporting the results of this study are available within the article and its supplemental material. Additional data, if required, are available with the corresponding author on reasonable request.

## SUPPLEMENTAL MATERIAL

Fig. S1 iai.00697-25-s0001.tif CFU analysis of the antibacterial activity of MSC-CM against *S. aureus* and *P. aeruginosa*.

Fig. S2 iai.00697-25-s0002.tif Time-kill kinetics of MSC-CM against *S. aureus* and *P. aeruginosa*.

Fig. S3 iai.00697-25-s0003.tif Disruption of pre-established biofilms by MSC-CM.

Supplemental material iai.00697-25-s0004.tif Graphical abstract.

ASM does not own the copyrights to Supplemental Material that may be linked to, or accessed through, an article. The authors have granted ASM a non-exclusive, world-wide license to publish the Supplemental Material files. Please contact the corresponding author directly for reuse.
