# Supplementary figures and images for: Mesenchymal stem cell-derived conditioned medium demonstrates novel antibacterial effects in ocular bacterial infections
Source: Infect Immun. 2026 Apr 30;94(6):e00697-25. doi: 10.1128/iai.00697-25 (PMC13248654; doi:10.1128/iai.00697-25)

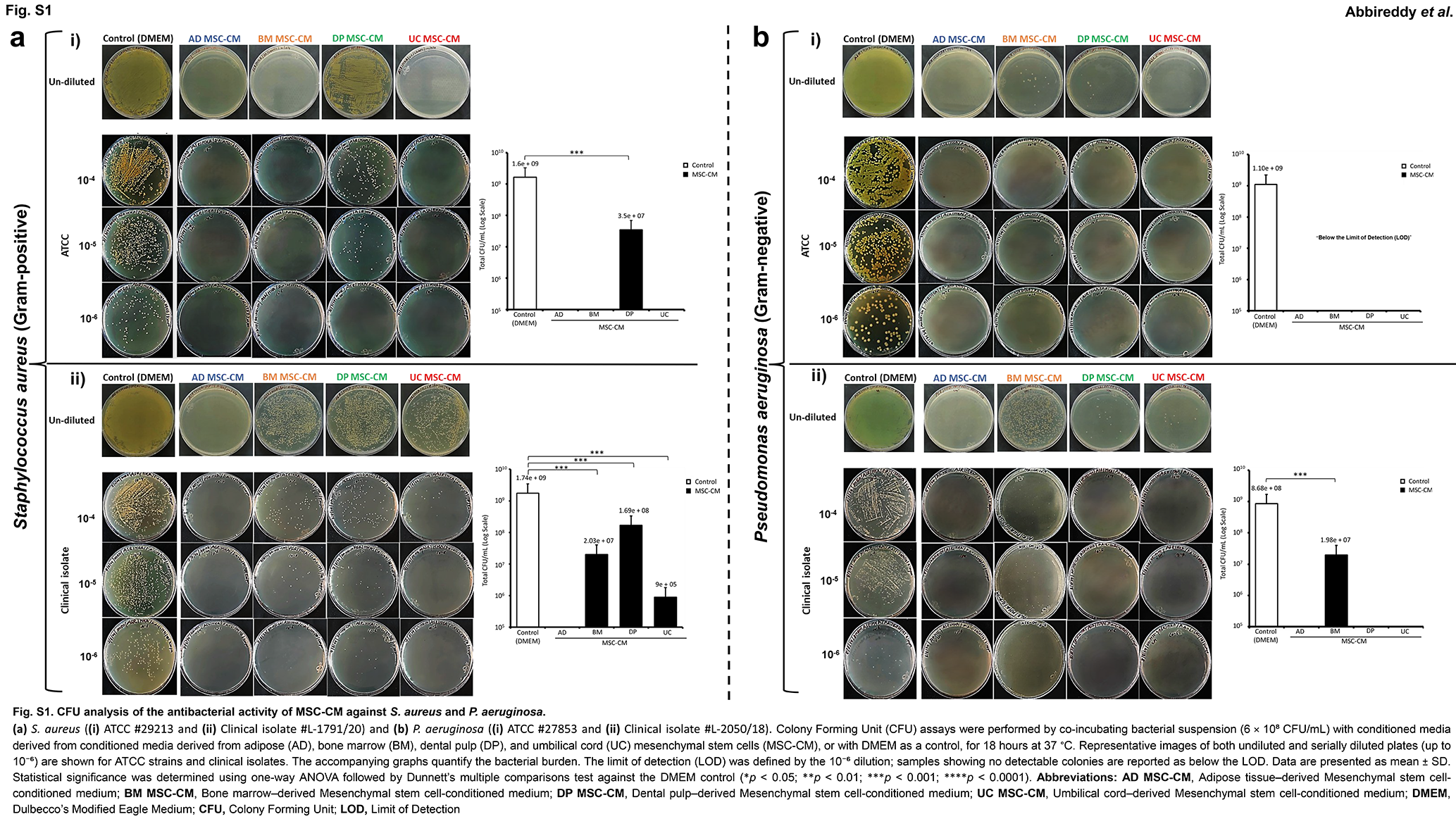

Supplement: Fig. S1 — CFU analysis of the antibacterial activity of MSC-CM against S. aureus and P. aeruginosa. [file iai.00697-25-s0001.tif]

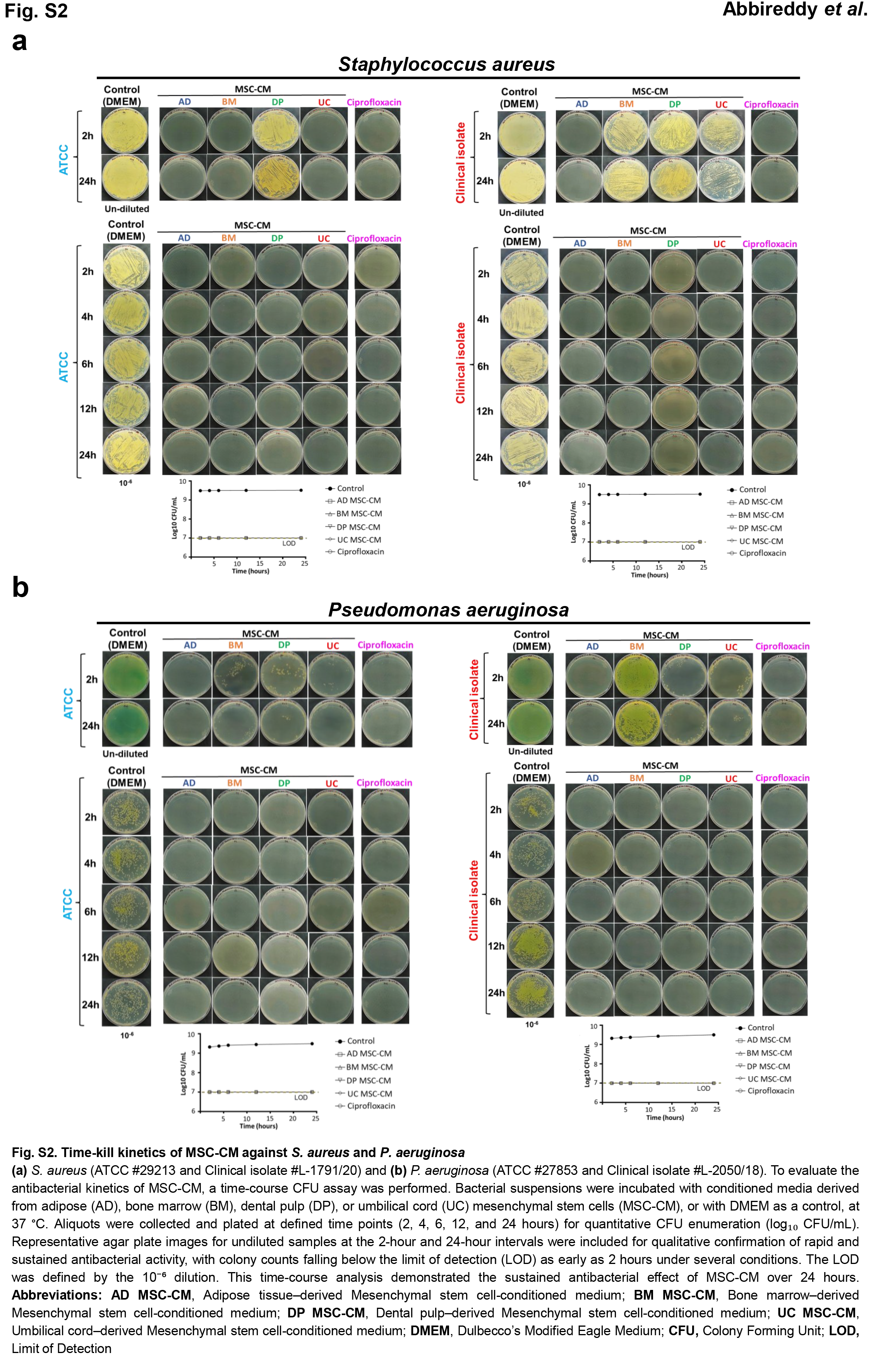

Supplement: Fig. S2 — Time-kill kinetics of MSC-CM against S. aureus and P. aeruginosa. [file iai.00697-25-s0002.tif]

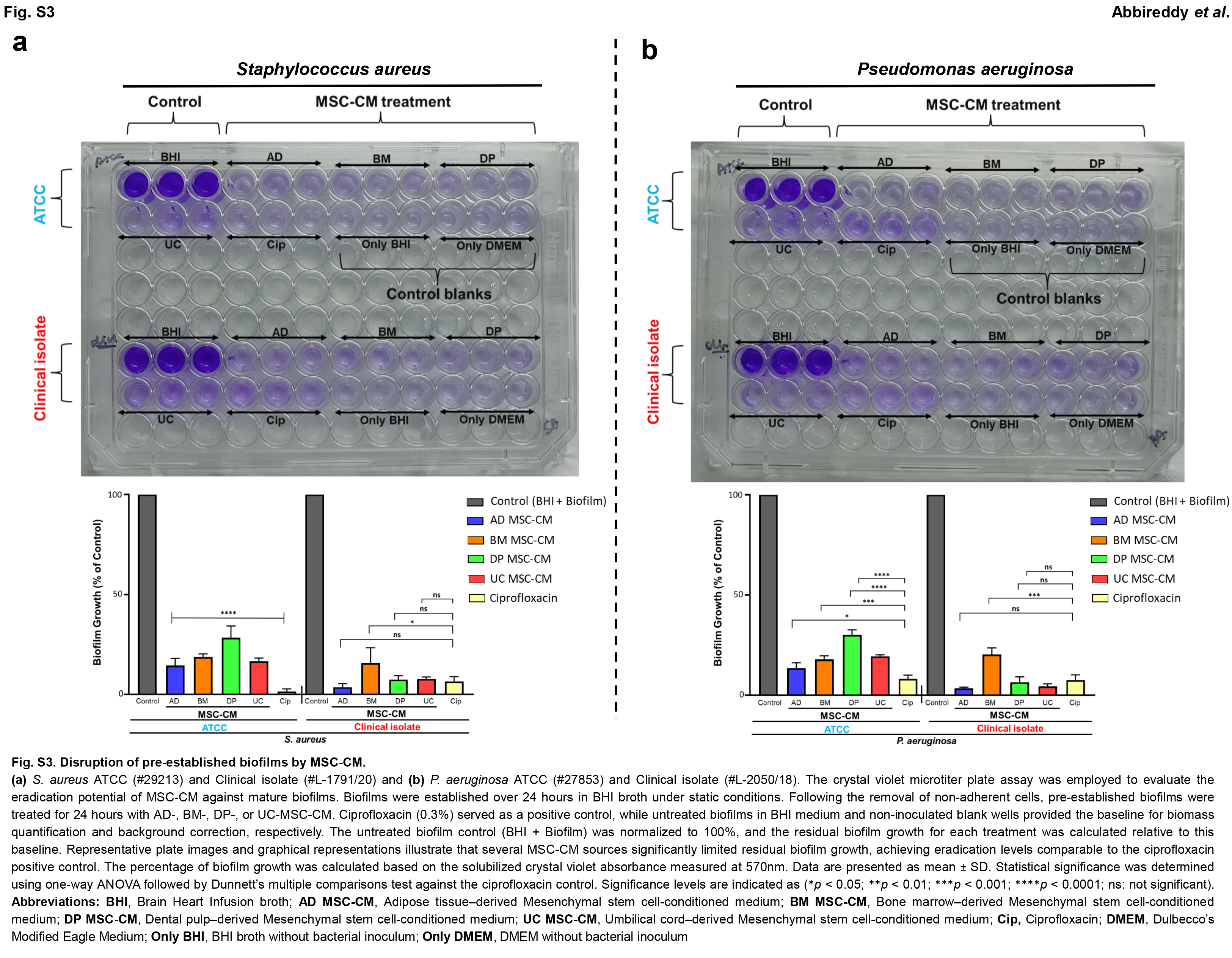

Supplement: Fig. S3 — Disruption of pre-established biofilms by MSC-CM. [file iai.00697-25-s0003.tif]

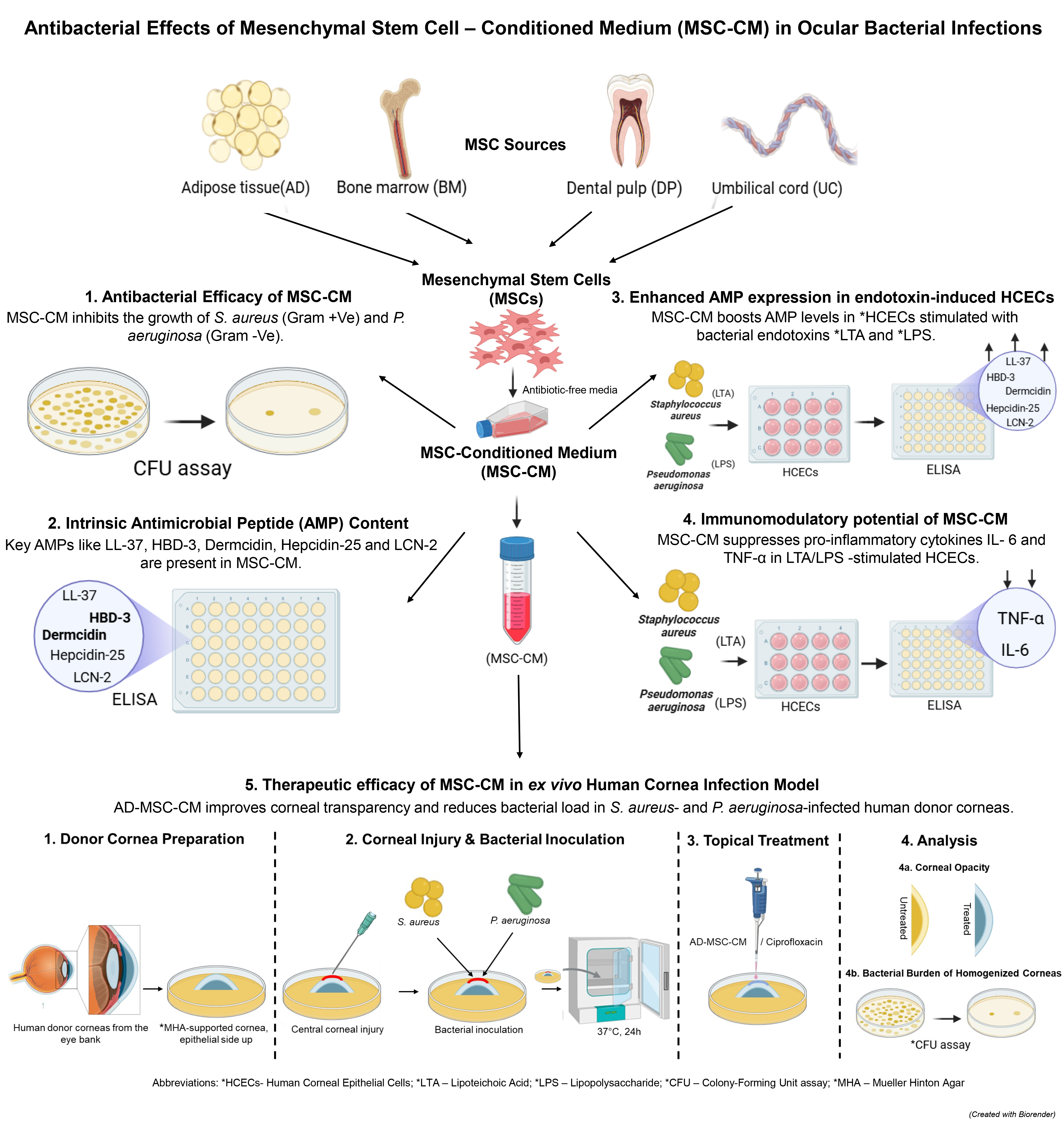

Supplement: Supplemental material — Graphical abstract. [file iai.00697-25-s0004.tif]
